# Conduction Cooling and Plasmonic Heating Dramatically Increase Droplet Vitrification Volumes for Cell Cryopreservation

**DOI:** 10.1002/advs.202004605

**Published:** 2021-04-10

**Authors:** Li Zhan, Shuang‐Zhuang Guo, Joseph Kangas, Qi Shao, Maple Shiao, Kanav Khosla, Walter C. Low, Michael C. McAlpine, John Bischof

**Affiliations:** ^1^ Department of Mechanical Engineering University of Minnesota Minneapolis MN 55455 USA; ^2^ Center for Advanced Technologies for the Preservation of Biological Systems (ATP‐Bio) University of Minnesota Minneapolis MN 55455 USA; ^3^ School of Materials Science and Engineering Sun Yat‐sen University Guangzhou 510275 China; ^4^ Department of Neurosurgery University of Minnesota Minneapolis MN 55455 USA; ^5^ Stem Cell Institute University of Minnesota Minneapolis MN 55455 USA; ^6^ Department of Biomedical Engineering University of Minnesota Minneapolis MN 55455 USA

**Keywords:** cell therapy, conduction cooling, cryopreservation, droplet vitrification, plasmonic laser heating

## Abstract

Droplet vitrification has emerged as a promising ice‐free cryopreservation approach to provide a supply chain for off‐the‐shelf cell products in cell therapy and regenerative medicine applications. Translation of this approach requires the use of low concentration (i.e., low toxicity) permeable cryoprotectant agents (CPA) and high post cryopreservation viability (>90%), thereby demanding fast cooling and warming rates. Unfortunately, with traditional approaches using convective heat transfer, the droplet volumes that can be successfully vitrified and rewarmed are impractically small (i.e., 180 picoliter) for <2.5 m permeable CPA. Herein, a novel approach to achieve 90–95% viability in micro‐liter size droplets with 2 m permeable CPA, is presented. Droplets with plasmonic gold nanorods (GNRs) are printed onto a cryogenic copper substrate for improved cooling rates via conduction, while plasmonic laser heating yields >400‐fold improvement in warming rates over traditional convective approach. High viability cryopreservation is then demonstrated in a model cell line (human dermal fibroblasts) and an important regenerative medicine cell line (human umbilical cord blood stem cells). This approach opens a new paradigm for cryopreservation and rewarming of dramatically larger volume droplets at lower CPA concentration for cell therapy and other regenerative medicine applications.

## Graphical abstract

A novel droplet vitrification system demonstrates high post thaw cell viability (90–95%) with reduced cryoprotectant concentration (i.e., 20%) and improved vitrification throughput (i.e., ≈1000x) using larger (1–4 μL) droplet volumes. Conductive cooling and plasmonic heating approaches are employed and characterized thoroughly to substantially improve the cooling and warming rates compared to traditional convective heat transfer approaches.

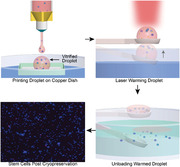

## 1 Introduction

Cell therapies using engineered T‐cells, stem cells, hepatocytes, and other primary cell types hold great potential to revolutionize treatments for cancer, neurodegenerative disorders, spinal cord injuries, diabetes, and many other ailments.^[^ ^1^, ^2^, ^3^ ^]^ To achieve the off‐the‐shelf availability and allow easy transport of these cell products, successful cryopreservation with high recovery (i.e., >90%) is an important cornerstone as dead cells will complicate direct in vivo use.^[^ ^4^, ^5^, ^6^, ^7^, ^8^ ^]^ Cryopreservation stops cellular metabolism by cooling the cells in liquid nitrogen (LN_2_) to −196 °C, stabilizing the cells for long‐term storage. In this state, the cells will also avoid undesired differentiation and genetic drift which occurs during proliferation through extended culture processes.^[^ ^9^, ^10^, ^11^ ^]^ To avoid lethal ice formation during cryopreservation, cryoprotectant agent (CPA) is employed to penetrate into the cell to replace intracellular water content, and/or remain outside of the cell to mitigate extracellular ice formation.^[^ ^9^, ^10^ ^]^ In general, cell membrane permeable CPAs that are commonly used include ethylene glycol (EG), propylene glycol (PG), and dimethyl sulfoxide (DMSO), while sugars such as sucrose and trehalose, or polymers including polyethylene glycol (PEG) and dextrans are largely non‐permeable.^[^ ^12^, ^13^ ^]^ The CPA toxicity, cooling rate, and warming rate are all important and interrelated factors impacting the cryopreservation outcomes.^[^ ^14^, ^15^, ^16^, ^17^ ^]^ For example, the common goal of reducing intracellular CPA concentration (i.e., lowering toxicity), requires high cooling and warming rates to minimize lethal ice formation to achieve a high survival rate.^[^ ^17^, ^18^, ^19^ ^]^

Slow freezing is the traditional cryopreservation method after cells are equilibrated with low permeable CPA concentration (i.e., 1.4 M DMSO) in a cryovial. Slow cooling rate (i.e., 1°C min^−1^) allows the growth of extracellular ice crystals which exclude CPA molecules thereby raising the CPA concentration around cells. This then causes cell dehydration which increases the concentration of the intracellular CPA. The high CPA concentration favors vitrification during cooling, thereby reducing lethal intracellular ice formation. With this approach, viable cells eventually reside in a much smaller glassy phase between larger extracellular ice crystals. Rewarming is then traditionally performed by agitating the cryovial in a 37 °C water bath (i.e., ≈100°C min^−1^) until all ice disappears. Despite its wide use on immortalized cell lines, using slow freezing to achieve high viability in more sensitive primary cell types such as stem cells, hepatocytes, engineered T‐cells, and lymphocytes has largely failed.^[^ ^20^, ^21^, ^22^, ^23^, ^24^ ^]^ Indeed, the extreme osmotic and mechanical stress, CPA toxicity, and recrystallization during rewarming remain essential challenges for the slow freezing method, especially in primary cell lines that are of increasing use in regenerative medicine.^[^ ^20^, ^25^ ^]^

Vitrification, a direct transition process from liquid to glass phase by rapid cooling, can avoid both intra‐ and extra‐cellular lethal ice formation.^[^ ^26^, ^27^, ^28^ ^]^ Vitrification has shown superior outcomes than slow freezing in cryopreservation of numerous biomaterials.^[^ ^29^, ^30^, ^31^, ^32^ ^]^ To achieve successful vitrification‐based cryopreservation, samples need to be cooled and rewarmed at a rate above the critical cooling rate (CCR) and critical warming rate (CWR) of the chosen CPA solution, respectively. The lower the CPA concentration, the higher the CCR and CWR. In addition, the CWR is usually 100–1000‐fold higher than the CCR to avoid ice formation (i.e., devitrification) upon rewarming.^[^ ^33^ ^]^

**Scheme 1:**
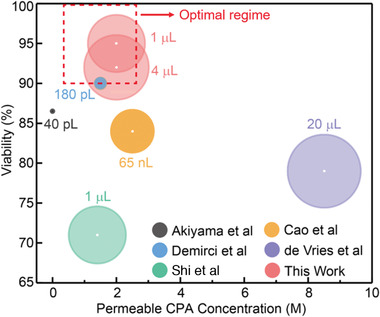
Comparison of droplet vitrification based cell cryopreservation methods. Droplet size, permeable CPA concentration, and post cryopreservation viability were plotted. The size of the sphere (relative log scale) represents droplet size. Larger droplet size, lower permeable CPA concentration, and higher viability are desired (i.e., boxed area in red).

To achieve vitrification, many groups have focused on the use of droplets which coupled with convection can achieve rapid cooling rates due to the large surface area to volume ratio.^[^ ^34^ ^]^ As shown in **Table** **1** and **Scheme** **1**, all the previous droplet vitrification studies used convective heat transfer for cooling and/or rewarming. This poses a fundamental barrier that limits the size of the droplets to achieve a high post rewarming viability (i.e., >90%). Specifically, the achievable cooling and warming rates are inversely proportional to the droplet size. In order to use lower CPA concentration, the droplet size needs to be further decreased, otherwise, the viability will be compromised due to ice formation caused by insufficient cooling and warming rates. For example, using <2.5 m permeable CPA, Demirci et al. and Akiyama et al. used 180 pL and 40 pL cell encapsulated droplets for vitrification and reported 90% and 87% post rewarming viability, respectively.^[^ ^34^, ^35^ ^]^ As the droplet volume increased to 65 nL (Cao et al.) and 1 µL (Meng et al.), the viability dropped to 84% and 71% respectively.^[^ ^25^, ^36^ ^]^ In order to vitrify larger sized droplet (i.e., 14–65 µL), de Vries et al. required much higher CPA concentrations (i.e., 8.5 m) to obtain a viability of only 79%.^[^ ^20^ ^]^ In addition, the broad distribution of droplet size led to inconsistency in the cooling and warming rate, as well as variability in the viability post cryopreservation.^[^ ^20^ ^]^ Further, convective cooling by printing the droplet directly into LN_2_ is hampered by the Leidenfrost effect.^[^ ^37^, ^38^ ^]^ When a droplet hits the LN_2_ surface, LN_2_ starts to boil and forms a nitrogen vapor layer around the droplet. This vapor layer has poor thermal conductivity thereby reducing directly cooling from the LN_2_ and causing the droplet to float on the surface, where both effects substantially slow the cooling. Although droplet size can be reduced to picoliter scale for rapid cooling in LN_2_, the vitrification throughput is low in the single µL min^−1^ range.^[^ ^34^, ^35^ ^]^ This reduces scalability when liters of cell suspension need to be vitrified for clinical use. As shown in the Scheme 1 (i.e., the boxed area in red), the ideal droplet vitrification method for cell therapy applications should provide a high post cryopreservation viability (≥90%) using low permeable CPA concentration (≤2.5 m), and in a large sized droplet (≥1 µL). Nonetheless, the previous convective heat transfer based droplet vitrification methods failed to achieve this due to the underlying heat transfer constraints.

**Table 1:** Droplet vitrification‐based cryopreservation methods

Droplet size	CPA used	Vitrification throughput	Cooling rate [°C min^−1^]	Warming methods	Warming rate [°C min^−1^]	Viability	Reference
40 pL	CPA free	4.8 µL min^−1^	2.2 × 10^6^	convective	9 × 10^6^	87%	Akiyama et al., 2019^[^ ^35^ ^]^
180 pL	1.5 m PG + 0.5 m trehalose	9 µL min^−1^	N/A	convective	N/A	90%	Demirci et al., 2007^[^ ^34^ ^]^
65 nL	1 m EG + 1.5 m PG + 1 m trehalose	10 µL min^−1^	N/A	convective + MIH	N/A	84%	Cao et al., 2019^[^ ^25^ ^]^
1 µL	1.4 m DMSO	N/A	1.1 × 10^3^	convective	6.9 × 10^3^	71%	Shi et al., 2015^[^ ^36^ ^]^
≈14–65 µL	8.5 m DMSO	4 mL min^−1^	9.6 × 10^2^	convective	N/A	79%	de Vries et al., 2018^[^ ^20^ ^]^
1 µL	2 m PG + 1 m trehalose	0.6 mL min^−1^	1.75 × 10^4^ ^a)^	laser warming	≈7.6 × 10^5^–4.4 × 10^6^	95%	This work
4 µL		2.4 mL min^−1^	9 × 10^3^ ^a)^		≈3.5 × 10^5^–4.4 × 10^6^	92%	

^a)^Measured cooling rate at the top of the droplet (i.e., slowest cooling rate within the droplet, details in Figure 3)

**Figure 1:**
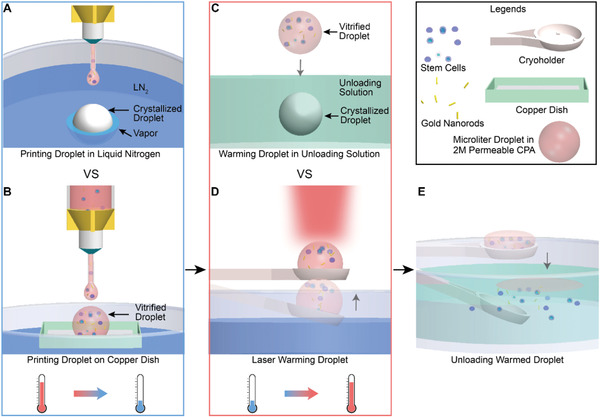
Schematics of microliter sized droplet vitrification using 2 m permeable CPA. A) Gold nanorods (GNRs) and biomaterials (i.e., stem cells) were first mixed with CPA for subsequent droplet printing. When a droplet was printed directly to LN_2_, the instantaneous boiling of LN_2_ created a “vapor blanket”, known as the Leidenfrost effect. This nitrogen vapor held the droplet floating, slowed down the cooling rate, and eventually led to crystallization. B) Droplet was printed onto a cryogenic copper dish floating on the LN_2_. Faster cooling rate through heat conduction can be achieved by avoiding the Leidenfrost issue, leading to successful vitrification. C) For convective rewarming, a vitrified droplet was directly dropped to the CPA unloading solution. Devitrification occurred due to slow warming rate. D) For laser warming, a vitrified droplet on the cryoholder was brought up to the millisecond pulse laser beam. E) After laser warming, biomaterials were released into CPA unloading solution. 2 m propylene glycol (PG) and 1 m trehalose was used as the CPA in this study.

This work then summarizes our use of conduction and plasmonics to overcome previous convective approaches as shown in **Figure** **1**. More specifically, we successfully achieved >90–95% post warming viability in microliter sized droplets with 2 m permeable CPA and 1 m trehalose using human umbilical cord blood stem cells (UCBSC), exceeding the capabilities of traditional convective heat transfer based methods (Scheme 1). Specifically, we substantially improved droplet cooling rates by printing onto a cryogenic copper dish floating on LN_2_. During the printing process, the droplet size can be controlled by the tip diameter, printing pressure, and time of the 3D printer. The direct contact with a cooled substrate at −196 °C facilitated a more efficient heat transfer by conduction versus convection, leading to successful vitrification in a microliter sized droplet. Non‐permeable CPA (i.e., 1 m trehalose) was added to the permeable CPA (i.e., 2 m PG) to reduce the CCR and CWR. We measured the cooling rates within the droplet and confirmed the vitreous status via X‐ray diffraction and Raman spectroscopy. Further, to overcome the slow warming rates (i.e., ≈10^4^ °C min^−1^) in traditional convective warming methods, we achieved ultra‐rapid warming using gold nanorod (GNRs) induced laser heating method.^[^ ^18^, ^39^ ^]^ Specifically, the GNRs were mixed in the CPA prior to vitrification and absorbed the laser energy to heat the vitrified droplet at a rate of ≈10^6^ °C min^−1^. We thoroughly characterized the laser warming of the droplet through Monte Carlo and heat transfer modeling, combined with high‐speed camera experiments.

## 2 Results and Discussion

### 2.1 Droplet Printing and Vitrification

**Figure 2:**
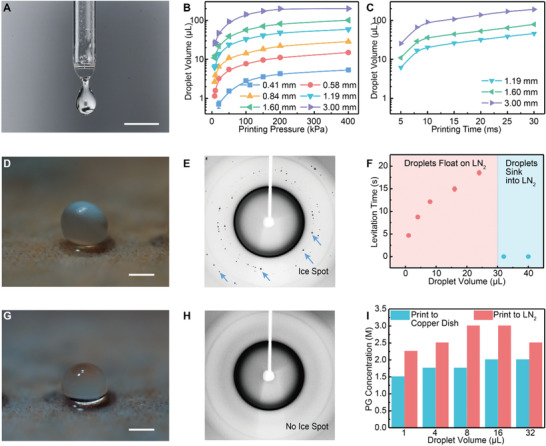
Microliter CPA droplet printing and vitrification. A) Image of printed droplet next to the printing tip, captured by camera with a high‐speed flashlight. B) The effect of printing pressure and printing tip diameter on the droplet volume (*n* = 20). Printing time was 5 ms. C) The effect of printing time (i.e., pressure pulse duration) and printing tip diameter on the droplet volume (*n* = 20). Printing pressure was 8 kPa. D) Image of a crystallized droplet (4 µL) when the droplet was printed directly into LN_2_. E) Representative X‐ray diffraction pattern of a crystallized droplet. The dark ring represents the tubing material used to hold the droplet for measurement. F) Levitation time of droplet on the surface of LN_2_ increased with increasing droplet volume (*n* = 10). But for large droplet size (i.e., 32 and 40 µL), droplet sank directly into LN_2_. G) Image of a vitrified droplet (4 µL) when the droplet was printed onto a cryogenic copper dish. H) Representative X‐ray diffraction pattern of a vitrified droplet. I) Required PG concentration for vitrification using different droplet sizes and cooling methods (*n* = 5). In (B,C,F), data points represent mean ± s.d.. Error bars are included, but may not be visible. Scale bars are 1 mm.

First, we investigated our ability to print droplets of well‐controlled size based on the printing parameters. To print microliter sized droplets, the syringe with a printing tip was loaded with CPA solution (2 m PG + 1 m trehalose) and connected to a pressure dispenser. Each pressure pulse generated by the pressure dispenser printed one droplet through the printing tip (**Figure** **2**A; MovieS1, Supporting Information). The volume of the printed droplet can be tuned by changing the tip diameter, pressure, and time (i.e., pressure pulse duration). As shown in Figure 2B,C, the droplet volume increased from 0.62 to 175 µL with increasing printing pressure, time, and tip diameter. The minimum volume of the droplet that can be printed increased as the tip diameter increased (Figure 2B). In addition, a high‐speed camera was used to record the printing process and revealed that the time to form one droplet is close to the applied printing time (Figure S1, Supporting Information). For instance, by printing ten droplets per second, we achieve 2.4 mL min^−1^ vitrification throughput when 4 µL droplets were printed. This is a >1000 fold improvement compared to the throughput of ≈µL min^−1^ when nano‐ or pico‐liter droplets were used in the previous droplet vitrification work (Table 1).

We then printed the droplet directly into LN_2_ to assess our ability to vitrify. The Leidenfrost effect led to a nitrogen vapor layer under the droplet that kept it floating. As the temperature of the droplet decreased, the vapor layer weakened and the droplet eventually sank into LN_2_. We found the levitation time is droplet size dependent. Above a critical size (i.e., ≥32 µL), droplets immediately sank into LN_2_ (Figure 2F). For small droplets, the levitation time increased from 4.7 ± 0.3 s to 18.5 ± 0.5 s as the droplet size increased from 1 to 24 µL. For a 4 µL droplet, direct printing into LN_2_ led to crystallization (Figure 2D). To overcome this Leidenfrost issue, we demonstrated successful vitrification by printing the droplet onto a cryogenic copper dish floating on LN_2_. A vitrified 4 µL droplet showed transparent appearance (Figure 2G). Indeed, direct contact with a cold substrate (i.e., −196 °C) allowed more efficient heat transfer to cool the droplet via conduction, compared to the convective heat transfer when a droplet was printed into LN_2_. However, we observed condensation on the copper surface over time, likely consisting of the mixture of LN_2_ and liquid oxygen (boiling point −183 °C). This cryogenic liquid accumulated in the copper dish and prohibited the direct contact between the droplet and the copper surface, which led to the Leidenfrost issue again. We addressed this by placing a wicking material on the copper surface so that the droplet can maintain direct contact with a cryogenic substrate. Wicking materials with two different microstructures were tested. One has larger porous openings (i.e., cleanroom wipe) and the other has minimal porous openings (i.e., Kimwipes and filter paper, Figure S2, Supporting Information). The vitrified droplets stuck into the cleanroom wipe and broke apart when we attempted to remove them. This indicated that part of the droplet infiltrated into the large pores before freezing occurred and remained inside afterwards. In the case of Kimwipes and filter paper, vitrified droplets can be easily removed (Figure S3, Supporting Information). The Kimwipes was placed on the copper dish for subsequent printing.

Further, we compared the minimal PG concentration required to achieve vitrification (i.e., transparent appearance) for printing into LN_2_ or the cryogenic copper dish. Various droplet sizes were tested, and 1 m trehalose was added in all CPAs. In all the droplet sizes, printing onto the copper dish required lower PG concentration (i.e., >20%) for vitrification compared to printing to LN_2_ (Figure 2I). When printed into LN_2_, the 32 µL droplet directly sank and cooled at a faster rate than the 16 µL droplet which floated, therefore requiring a lower PG concentration for vitrification (Figure 2I). The required PG concentration increased from 1.5 to 2 m as droplet size increased from 1 to 32 µL in the case of printing on the copper dish. Altogether, we selected 2 m PG + 1 m trehalose as the CPA for the following study as it can be vitrified in 32 µL droplets, which provided wide range of droplet sizes for continued characterization.

### 2.2 Characterization of the Vitrified State

X‐ray diffraction and Raman spectroscopy were used to examine the vitreous state of the droplet. A printed 4 µL droplet was removed from the copper dish after printing and held by a PTFE tubing under cold nitrogen stream (−170 ⁰C) for the X‐ray diffraction measurement (Figure S4, Supporting Information). “Ice spots” were identified in the X‐ray diffraction pattern of the droplet printed to LN_2_ (i.e., crystallized droplet, Figure 2D,E). The dark ring in Figure 2E,H represented the PTFE tubing material. The radial location of the “ice spots” indicated the interplanar spacing (i.e., d‐spacing) of the crystal structure. Crystallized droplet showed d‐spacing peaks at 2.6 Å, 3.4 Å,3.6 Å, and 3.9 Å which are associated with hexagonal ice crystals (Figure S5, Supporting Information).^[^ ^40^, ^41^ ^]^ The CPA droplets printed to the cryogenic copper dish showed a broad peak around 3.6 Å, corresponding to the vitrified state reported in the literature (Figure 2H; Figure S5, Supporting Information).^[^ ^40^, ^41^ ^]^ In addition, we measured the Raman spectra of CPA and water droplets printed onto the copper dish. The peak of O—H stretching band (≈3100 cm^−1^) in CPA droplet shifts toward higher wavenumber compared to the water droplet (i.e., crystallized), suggesting that the CPA droplet is vitrified (Figure S6, Supporting Information).^[^ ^35^ ^]^ Although X‐ray diffraction and Raman spectroscopy cannot be easily used on every droplet, our measurements (n ≥ 3 for each case) suggest that the transparent appearance of the droplet in LN_2_ is a reasonably proxy to assess the vitreous state.

**Figure 3:**
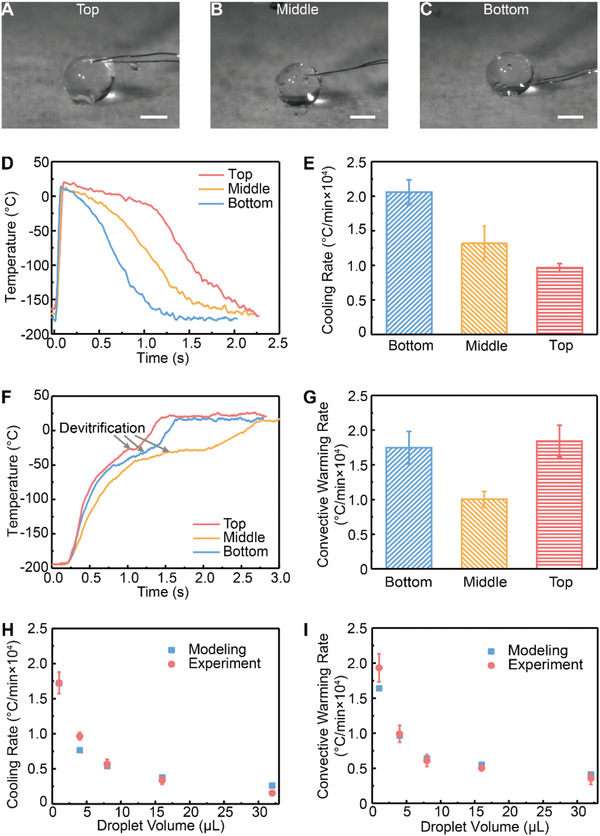
Measurement and modeling of temperature change rate within the droplets. A–C) A thermocouple was placed on the cryogenic copper dish at various distances from the surface. Droplet (4 µL) was printed onto the thermocouple such that the thermocouple was located at the bottom, center, or top of the droplet. D) Representative temperature profile recorded by the thermocouple during droplet cooling. E) Cooling rates at the bottom, middle, and top of the droplets (*n* = 5). Temperature zone from −20 °C to −140 °C was used to calculate the cooling rate. F) With thermocouple located within the vitrified droplet, temperature profile during convective warming was recorded. G) Convective warming rates at the bottom, middle, and top of the droplets (*n* = 5). Temperature zone from −20 °C to −140 °C was used to calculate the warming rate. In (D–G), droplet volume is 4 µL. H) Comparison of simulated and experimental cooling rate at the top of various sized dropletc (*n* = 5). I) Comparison of simulated and experimental convective warming rate at the middle of various sized droplet (*n* = 5). Scale bars are 1 mm. Data points represent mean ± s.d..

To measure the cooling rate, we printed the droplet onto a thermocouple (50 µm wire diameter) located at a different height from the surface of cryogenic copper dish. **Figure** **3**A–C shows the images of a thermocouple at different locations inside a 4 µL droplet. The temperature profile at the bottom, center, and top of the droplet was recorded by the thermocouple. As shown in Figure 3D, the temperature of the thermocouple was initially below −160 °C, and quickly increased to above 10 °C after being in contact with the printed 4 µL droplet, followed by a decline that reflected the cooling profile of the droplet. Cooling of the droplet was achieved by two driving forces including conduction through droplet–copper dish interface and natural convection between droplet and cold nitrogen vapor. For the bottom and middle locations of the droplet, linear temperature decline at one cooling rate was recorded. On the other hand, the top location of the droplet went through the cooling process with two different slopes (i.e., rates) at different times (Figure 3D). The first segment has a slower cooling rate (i.e., before 1.2 s in Figure 3D), due to the less efficient convective cooling by the surrounding cold nitrogen vapor. During this time, the top of the droplet did not “sense” the existence of the cold copper dish as it takes time for heat to diffuse across the droplet. The height (*H*) of a 4 µL droplet on copper dish is 1.8 mm (Figure 2). Assuming the droplet has thermal properties of vitrified CPA at ‐50 °C (average temperature of top, middle, and bottom location at 0.6 s), and the thermal diffusivity (*α*) is 10^−6^ m^2^ s^−1^.^[^ ^39^, ^42^ ^]^ The heat diffusion time across the droplet can be estimated as *t = H^2^ / 2α* = 1.62 s, which is comparable to the 1.2 s observed experimentally. After 1.2 s, heat conduction started to facilitate a faster cooling rate at the top of the droplet (Figure 3D). In addition, heat transfer simulations also show a two‐segment cooling profile at the top of the droplet (Figure S7, Supporting Information). For the 4 µL droplet, Figure 3E showed the cooling rates calculated using temperature zone from −20 °C to −140 °C. Specifically, the cooling rates at the bottom, middle, and top of the droplet were 2.1 ± 0.2 × 10^4^, 1.3 ± 0.3 × 10^4^, and 0.96 ± 0.05 × 10^4^ °C min^−1^, suggesting more than a twofold difference in cooling rates between bottom and top locations.

Various sized droplets ranging from 1 to 32 µL were printed onto the cryogenic copper dish and successfully vitrified (Figure S6, Supporting Information). Cracking occurred when a 64 µL droplet was printed, due to thermal stress built up within the droplet over the differential cooling process. Simulated temperature distribution within the droplet also suggested that the larger the droplet, the longer the time that a droplet was subject to the thermal gradient (i.e., the bottom versus top location, Figure S8, Supporting Information). As shown in Figures S3D and S7B, Supporting Information, the thermal gradient between the top and bottom locations first increased due to different heat transfer mechanisms (i.e., conduction for the bottom location and natural convection for the top location). After the top location “sensed” the cold copper dish and cooled by conduction, the thermal gradient decreased. Thus, for a larger sized droplet, the time for heat to diffuse from the bottom to top location increased, leading to a higher risk for cracking. Importantly, this well‐controlled (i.e., CPA concentration, temperature profile, spatial dimension) conduction cooling method can provide a simple quantitative testbed to understand the thermo‐mechanical interaction of cracking during vitrification. Further, we selected the top location in various sized droplets to compare the measured and simulated cooling rates. The modeling results matched well with the experimental measurements (Figure 3H). With increasing droplet size, the cooling rates dropped rapidly. For instance, for 1, 8, and 32 µL droplets, cooling rates at the top location were measured as 17 ± 2 × 10^3^, 5.7 ± 0.6 × 10^3^, and 1.5 ± 0.3 × 10^3^ °C min^−1^, respectively.

### 2.3 Convective Warming

**Figure 4:**
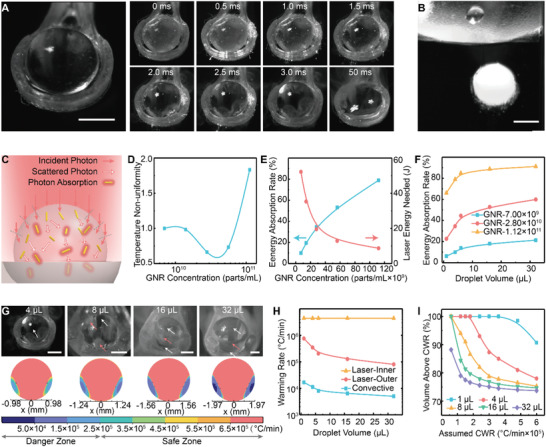
Rewarming of the microliter vitrified droplet. A) Left: image of a 4 µL vitrified droplet on the cryoholder brought out of LN_2_. Right: after the remaining LN_2_ around the droplet evaporated, the sequential snapshots of the droplet during and after a 2.5 ms laser pulse. No apparent ice formation or boiling was observed during laser warming. B) A 4 µL vitrified droplet was dropped into the CPA unloading solution for convective warming. Apparent ice formation (i.e., white color) was noted. C) Schematics of Monte Carlo modeling for GNR‐induced laser warming within the droplet. D) Simulated temperature non‐uniformity (i.e., standard deviation of the temperature) of the laser warmed droplet with various GNR concentrations, normalized by GNR concentration of 7 × 10^9^ parts mL^−1^. E) Simulated energy absorption rate (blue), the ratio between absorbed energy by the droplet and total incoming laser energy. Measured laser energy (red) that is required to rewarm the droplet to complete melt. F) Simulated energy absorption rate in various droplet sizes with different GNR concentrations. G) Top: images of various sized droplets right after the 2.5 ms laser pulse. White arrows indicate liquid area and red arrows for ice area. Bottom: Simulated warming rate distribution within various sized droplet. Warming rate lower than 2.5 × 10^5^ °C min^−1^ indicates the danger zone (i.e., devitrification), higher than 2.5 × 10^5^ °C min^−1^ indicates the safe zone. H) Simulated average warming rates of various sized droplets using laser and convective warming methods. For laser warming, the droplet was separated into inner area (i.e., more laser access) and outer area (i.e., less laser access due to refraction). I) Simulated volume percentage of the droplet with warming rate above a certain assumed critical warming rate (CWR). Multiple droplet sizes were plotted. Scale bars are 1 mm.

To rewarm vitrified CPA droplets, traditional convective warming was performed by depositing the droplets into the water at room temperature. For example, the 4 µL droplet turned a white color from initial transparent appearance, suggesting ice formation (i.e., devitrification) due to slow warming rates (**Figure** **4**). To measure the convective warming rate, we used the vitrified droplets that were printed onto the thermocouple shown in Figure 3A–C. For example, thermocouple recorded the warming temperature profile at the bottom, middle, and top locations within the 4 µL droplet (Figure 3F). A plateau at subzero temperatures was observed in the measured warming curve, indicating devitrification. For the 4 µL droplet, the warming rate at the bottom, middle, and top locations were 1.7 ± 0.2 × 10^4^, 1 ± 0.1 × 10^4^, and 1.8 ± 0.2 × 10^4^°C min^−1^, respectively. In addition, we compared the measured and simulated convective warming rates using the middle location in different droplet sizes (Figure 3I). With similar warming rates reported by the experiments and modeling, the convective warming rates dropped for large sized droplet. For instance, the measured convective warming rates at the middle location are 19.3 ± 2 × 10^3^, 6.1 ± 0.8 × 10^3^, and 3.5 ± 0.8 × 10^3^ °C min^−1^, for 1, 8, and 32 µL droplets, respectively. Devitrification was noted in all droplet sizes (i.e., 1–32 µL) during convective warming.

### 2.4 Laser Warming

To achieve rapid warming and avoid devitrification, we employed GNRs induced laser warming. The GNRs were added in the CPA solution prior to droplet printing. The vitrified droplet was placed onto a customized cryoholder in LN_2_ and brought under the laser beam for rewarming. The laser beam diameter was set to 4.4 mm. The transverse energy distribution profile of the laser beam was measured using the knife‐edge method (Figure S9, Supporting Information). We showed the laser energy is uniformly distributed within the beam (i.e., a top‐hat beam instead of Gaussian beam). A high‐speed camera was used to monitor the droplet during laser warming at 4000 frames per second. Underheating (i.e., devitrification) and overheating (i.e., boiling) can be visualized from the camera (Figure S10, Supporting Information). With optimal GNR concentration and laser energy, the droplet can be rewarmed within a single ms laser pulse while avoiding underheating or overheating. For example, Figure 4A showed the images of a 4 µL droplet at different times before, during, and after the 2.5 ms laser pulse. When brought out of LN_2_ to the laser beam, the droplet appeared transparent and was surrounded by some residual LN_2_ (Figure 4A). We fired the laser pulse after confirming all LN_2_ evaporated (Figure 4A, *t* = 0 ms). The droplet was successfully rewarmed to the liquid state at the end of laser pulse (i.e., *t* = 2.5 ms), and eventually spread on the cryoholder (i.e., *t* = 3 ms, 50 ms). Visualization by a high speed camera provides the direct evidence of ice‐free rapid rewarming of a spherical vitrified droplet (Movie S2, Supporting Information).

We used Monte Carlo simulation to trace the interactions between GNRs and laser within the vitrified droplet. Specifically, inside the droplet, the photons (i.e., laser) will be either scattered and change propagation direction, or absorbed and converted to heat by the GNRs (Figure 4C). Monte Carlo simulation provides the spatial distribution of the specific absorption rate (SAR, W m^−3^) generated by the laser–GNRs interaction.^[^ ^43^ ^]^ This SAR profile was then imported into a heat transfer model to simulate the temperature distribution of the droplet. In addition, Monte Carlo simulation included the refraction at the air–droplet interface (Figure 4C). We explored the impact of GNR concentration, laser energy, and droplet size on the rewarming using the modeling.

With different GNR concentrations ranging from 7 × 10^9^ to 1.1 × 10^11^ particles per mL, we first examined the temperature non‐uniformity of the droplet right after the laser pulse (Figure S11, Supporting Information). Indeed, the spherical shape of the droplet serves as a lens to focus the laser beam within the droplet. Therefore, part of the droplet will not be rewarmed by the laser directly, but by the conduction via the laser warmed region after the laser pulse. In Figure S11, Supporting Information, as the GNR concentration increases, the “hot zone” (i.e., localized high temperature area) moves from the bottom to the top of the droplet. Specifically, with low GNR concentration, the laser can penetrate deeper into the droplet and will eventually be reflected and focused on the bottom, resulting in localized high temperature. However, with high GNR concentration, most of the laser energy is absorbed by the GNRs on the top of the droplet. We evaluated the temperature non‐uniformity within the droplet by calculating the standard deviation of the temperature within the droplet. This was then normalized by the value for GNR concentration of 7 × 10^9^ particles per mL. Figure 4D showed that GNR concentration of 2.8 × 10^10^ particles per mL provides the best temperature uniformity among those selected concentrations. Further, we assessed the energy absorption rate (the percentage of laser energy absorbed by the droplet) of different GNR concentrations for a 4 µL droplet. Figure 4E showed increasing energy absorption for higher GNR concentration through the modeling. The required laser energy to rewarm a vitrified 4 µL droplet to the completion of melt was evaluated using the high‐speed camera. Less laser energy is required to rewarm a droplet for higher GNR concentration due to a higher laser energy absorption rate (Figure 4E).

For different droplet size ranging from 1 to 32 µL, spherical droplet shape was used in the modeling for simplicity. We modeled the energy absorption rate using various GNR concentrations in Figure 4F. In the modeling, for a given GNR concentration, larger sized droplets have higher energy absorption rate. Additionally, we performed experiments to laser warm the vitrified droplets in different sizes using GNR concentration of 2.8 × 10^10^ particles per mL and 2.5 ms laser pulse. As shown in Figure 4G, after laser pulse, the 4 µL droplet showed no ice; but the 8 µL droplet had small amount of ice; 16 and 32 µL droplets showed an outer ice shell as captured by the high‐speed camera images (Movies S2 and S3, Supporting Information). Simulated warming rate distribution within the droplet suggested that for larger sized droplets, the danger of devitrification is larger (Figure 4G). This danger is most acute in the parts of the droplet with lower warming rates. Due to the lensing effect of the droplet curvature, only the “inner” portion of the droplet is rewarmed directly by laser (i.e., fast), the “outer” shell is rewarmed by conduction (i.e., sl ow). For a 4 µL droplet, the “outer” shell is thin and can be rewarmed faster than the CWR, resulting in ice free rewarming (Figure 4A). However, for 16 and 32 µL droplets, the “outer” shell is thicker and fails to be rewarmed fast enough, eventually leading to the formation of an ice shell although the inner layer achieves the liquid state. In Figure 4H, we compared the averaged warming rate of the “inner” and “outer” portion of the droplet during laser warming, as well as the averaged warming rate during convective warming for different droplet volumes. For laser warming, the “inner” portion of the droplet has similar warming rates (i.e., ≈4 × 10^6^ °C min^−1^) across different droplet sizes. The warming rate of the “outer” portion of the droplet decreased from 7.6 × 10^5^ to 2 × 10^5^ °C min^−1^ and 0.8 × 10^5^ °C min^−1^ as the droplet size increased from 1 to 8 µL and 32 µL, respectively. The convective warming rate is in the range of 0.5 × 10^4^ to 1.6 × 10^4^ °C min^−1^. Simulated warming rate using 1 µL droplet suggested that laser warming provided faster warming rate than convective warming at every location within the droplet (Figure S12, Supporting Information). To further characterize the laser warming, we evaluated the volume percentage of the droplet that has a warming rate above the assumed CWR value in Figure 4I. This could be used to estimate the viability after laser warming by assuming cells are dead if in the danger zone (i.e., warming rate below the CWR) and alive if in the safe zone (i.e., warming rate above the CWR). Importantly, the findings revealed by the modeling were supported by the experimental high speed videography (Figure 4). Altogether, the developed modeling approach helps to 1) understand the underlying physics and mechanisms of laser– GNR heating in a spherical vitrified droplet, and 2) optimize the key parameters including GNR concentration, laser power, and droplet size for successful rewarming.

### 2.5 Application to Cell Cryopreservation

**Figure 5:**
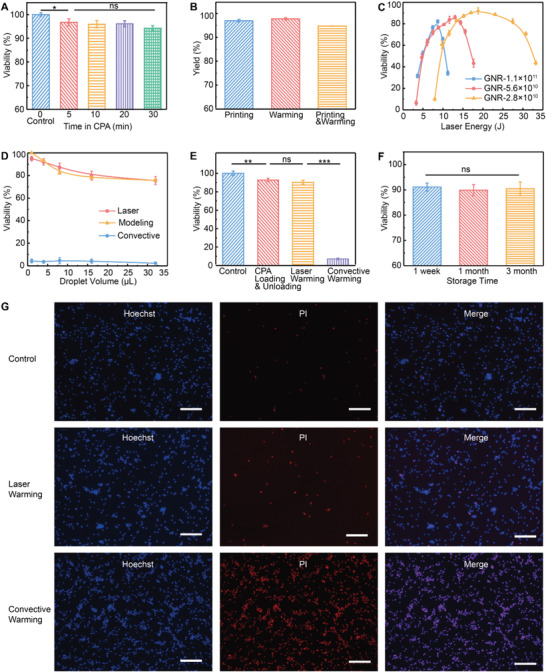
Cryopreservation of human dermal fibroblast (HDF) and human umbilical cord blood stem cells (UCBSCs). A) Cell viability after various exposure times to CPA solution at 4 °C. B) Yield of printing and laser warming. C) Post cryopreservation viability using different GNR concentrations and laser energies in 4 µL droplets. D) Experimental post cryopreservation viability using various droplet sizes and different warming methods. Simulated viability was calculated by the volume percentage of the droplet that has warming rate higher than 2.5 × 10^5^ °C min^−1^. E) Viability of control and various treatment groups including CPA loading and unloading, laser warming, and convective warming. F) Post cryopreservation viability after different storage time in LN_2_. G) Representative images of Hoechst (all cells) and propidium iodide (PI) (dead cells) for control group (top), laser warming group (middle), and convective warming group (bottom). A–D) HDFs. E–G) UCBSCs. Scale bars are 200 µm. Data points represent mean ± s.d. For (A,B,D–F), *n* = 5; for (C), *n* = 4. A paired one‐way ANOVA and Tukey‘s post hoc was used for statistical analysis. For the significance symbols: ns, *p* > 0.05, * *p* < 0.05, ** *p* < 0.01, *** *p* < 0.001.

Finally, we sought to apply our droplet printing, vitrification, and laser warming techniques for cell cryopreservation. We used a common cell line, human dermal fibroblast (HDF), to characterize and optimize the cryopreservation protocol, and then applied the protocol to human umbilical cord blood stem cells (UCBSCs). The UCBSCs are an important regenerative cell source which have been shown to ameliorate neurological deficits arising from ischemic brain injury via intravenous infusion.^[^ ^44^, ^45^ ^]^ HDF cell suspension was spun down to form a pellet, followed by removing the supernatant and adding ice‐cold CPA solution (2 m PG + 1m trehalose) with GNRs. Cells were incubated on ice for a range of times to test the CPA toxicity. To unload the CPA, we exposed the cells to 0.5 m sucrose as an osmotic buffer for 5 min on ice, then transferred them to fresh culture media for 5 min on ice. Cell viability was assessed by the membrane integrity assay Trypan blue. To reflect cell membrane integrity damage caused by CPA toxicity and cryoinjury during the cooling and rewarming processes, we reported the viability as the ratio of the intact cell percentage in recovered cell population post thaw to the intact cell percentage in initial unmanipulated cell population (**Figure** **5**). Figure 5A shows that increasing CPA loading time from 5 to 30 min did not affect the viability when 2 m permeable CPA concentration and low temperature (4°C) were used. This paves the road for processing large volumes of cell suspensions by continuous droplet printing in the future. During the uptake of CPA, water transport across the cell membrane occurs in response to the osmotic gradient, leading to cell volume change. This coupled intracellular CPA concentration and cell volume change can be modeled by the Kadeem–Katchalsky transport equations.^[^ ^20^ ^]^ By solving the K—K equations, we showed the intracellular PG concentration reached 1.4 m and the HDF cells remained dehydrated (i.e., shrunk) at the end of 5 min CPA loading (Figure S13, Supporting Information). The retention of the dehydrated state is attributed to the osmotic pressure exerted by the 1 m non‐permeable trehalose. In addition, simulated cell volume change indicated that the CPA unloading avoids over swelling (Figure S13, Supporting Information). Once loaded, the same printing parameters for CPA droplet were used for cell printing. This is justified since the cells occupy only 0.85% of the volume in the CPA solution, assuming cell density of 5 × 10^6^ cells per mL and cell diameter of 15 µm. To assess the yield of the printing process, cells were printed into a tube and the ratio of total amount of cells before and after printing was evaluated (Figure 5B). In addition, we collected the cell encapsulated droplets after printing onto the cryogenic copper dish and examined them under the X‐ray. The X‐ray diffraction pattern showed a broad peak, similar to the vitrified CPA droplet without any cells (Figure S5, Supporting Information).

To investigate the impact of laser warming on cell viability, cells were printed into 4 µL droplets with various GNR concentrations and rewarmed using different laser energies. A customized vacuum tubing device was used to transfer the vitrified droplets from copper dish to the cryoholder (Figure S14, Supporting Information). The transfer process was performed in LN_2_. The cryoholder with the vitrified droplet was then rapidly (0.3 s) brought out of LN_2_ using a customized cryojig to a preset location for laser warming. Rewarmed cells remained on the cryoholder and were quickly released to the unloading solution on ice. The yield of the cryopreservation process (i.e., printing and laser warming) was 94.8% (Figure 5B). This yield excluded cell loss due to the removal of supernatant after centrifugation. In Figure 5C, the correlation of viability versus laser energy showed an inverted V curve. Specifically, for a given GNR concentration, low viability was obtained for low laser energy (i.e., underheating) and high laser energy (i.e., boiling), with an optimal laser energy in between providing highest viability. This optimal laser energy for high viability is GNR concentration dependent. With higher GNR concentration, the optimal laser energy is lower, similar to the relationship shown in Figure 4E in the case of rewarming a vitrified CPA droplet. In addition, as a lower GNR concentration in the droplet leads to a lower laser energy absorption rate (Figure 4C), a broader span of laser energy in the inverted V curve of viability was presented in Figure 5C. Further, the optimal viability decreased as GNR concentration increased above 2.8 × 10^10^ particles per mL (Figure 5C). This is attributed to the non‐uniform temperature distribution within the droplet (i.e., localized hot zone) for higher GNR concentrations shown in Figure 4D; and Figure S11, Supporting Information. We then compared the cell viability in different sizes of droplets using corresponding optimal laser energies. In Figure 5D, viability remained <5% for convective warming due to devitrification. For laser warming, viability dropped from 95% to 75.5% when droplet size increased from 1 to 32 µL. The viability of various droplet sizes can be estimated by the volume percentage of the droplet that has a warming rate above the CWR of the CPA. However, the CWR of 2 m PG + 1 m trehalose is unknown. By comparing the measured viability (Figure 5D) with the simulated viability using various CWR values (Figure 4I), we estimated the CWR of 2 m PG + 1 m trehalose to be 2.5 × 10^5^ °C min^−1^ (Figure S15, Supporting Information). Indeed, the cell viability can be interpreted as a more accurate “sensor” to reflect the temperature within the droplet, leading to similar trends revealed by the modeling outcome shown in Figure 4. Indeed, laser warming can achieve a range of rapid warming rates (>10^5^ °C min^−1^) and cell viability can be used to reflect / identify the ice formation during the rewarming. Importantly, this new method will allow to estimate the CWR for certain CPA concentrations whose CWR were usually extrapolated from high CPA concentrations. Further, cells were stained with Hoechst / PI for visualization of the viability for control, convective rewarmed, and laser warmed cells (Figure S16, Supporting Information).

Using the optimal conditions for HDF cell cryopreservation, UCBSCs were loaded with 2 m PG + 1m trehalose + 2.8 × 10^10^ GNRs per mL for 5 min on ice. Droplets in 4 µL were then vitrified and laser warmed. Convective warming was also performed. Figure 5E presents the normalized viabilities (i.e., to control groups without any treatment) of control, CPA treated, laser warmed, and convective warmed cell. In the recovered cell population, laser warming provided 90.4 ± 2.2% viability compared to 92.7 ± 1.9% after CPA treatment (i.e., no cryopreservation) and 7.2 ± 0.9% after convective warming. In addition, a traditional slow freezing method using 10% DMSO was also performed and resulted in 79.3 ± 3.1% viability in the recovered cell population. To compare the post thaw viability with additional consideration of the cell loss due to experimental operation, laser warming (i.e., yield = 94.8 ± 0.2%) and slow freezing (i.e., yield = 100%) provide 86.6 ± 2.3% and 79.3 ± 3.1% viability, respectively (Figure S17, Supporting Information). This result indicates that our rapid cooling and warming techniques outperform the traditional slow freezing or convective droplet vitrification methods in terms of post thaw viability. Further, we stored those vitrified droplets with cells in LN_2_ for 1 week, 1 month, and 3 months. After reviving the cells using laser warming, comparable viabilities were observed for different storage time in LN_2_ (Figure 5F). Representative fluorescent images of Hoechst (i.e., all cells) / PI (i.e., dead cells) were presented in Figure 5G for control cells, laser warmed cells, and convective warmed cells.

## 3 Conclusion

In summary, we developed and thoroughly characterized the unique combination of various techniques (i.e., automated droplet printing, conduction cooling, and laser warming) to improve the ice‐free droplet vitrification based cryopreservation. Specifically, we used conduction and plasmonic laser warming techniques with the precision and automation of 3D printing to overcome convective heat transfer barriers in conventional droplet‐based vitrification cryopreservation. We successfully vitrified microliter sized droplets in low permeable CPA concentration (i.e., 2 m) + 1 m trehalose by printing onto a cryogenic substrate to achieve large volume (1000 x better than previous approaches) in mL min^−1^ (Table 1). We showed the permeable CPA concentration for vitrification can be reduced by >20% compared to the traditional convective cooling method by direct printing into LN_2_. X‐ray diffraction and Raman spectroscopy confirmed the vitreous status of the droplets. With GNRs mixed in the CPA solution, the vitrified droplets were successfully rewarmed by a laser pulse at a rate around 10^6^ °C min^−1^. Monte Carlo and heat transfer simulation predicted the optimal GNR concentration and laser energy for rapid and uniform rewarming in small droplet volume (i.e., <8 µL), validated by high‐speed videography. For larger droplet size (≥8 µL), the lensing effect of the droplet led to partial devitrification within the droplet, as predicted by the modeling. Furthermore, we applied the optimized conditions to print, vitrify, and rewarm HDF and stem cells in 1 and 4 µL droplets, resulting in >95 and 90% post cryopreservation viability, respectively as a proof‐of‐concept demonstration. Altogether, our approach demonstrated a novel path to cryopreserve cells in large droplets (i.e., ≥microliter) and lower permeable CPA concentration (i.e., 2 m) with higher viability (i.e., >90%). Future studies will focus on several directions including: (i) further optimization of the laser warming for larger droplets (>10 µL, Figure S18, Supporting Information); (ii) development of automatic laser warming to increase the throughput of rewarming; (iii) use of this approach on other cell therapy products such as pancreatic islets and adoptive T cell therapy or other.

## 4 Experimental Section

##### Cell Culture and Live/Dead Assay

Human dermal fibroblasts (HDFs) were cultured in Dulbecco's modified Eagle media (DMEM) that contained 10% fetal bovine serum (Thermo Fisher Scientific) and 1% penicillin streptomycin (Sigma) at 37 °C under 5% CO_2_. The HDFs were purchased from ATCC (PCS‐201‐012) and stored in a liquid nitrogen dewar that was maintained in the lab.

Human umbilical cord blood stem cells (UCBSC) were grown as previously described.^[^ ^45^ ^]^ Briefly, the cells were isolated from the mononuclear portion of human umbilical cord blood. They were obtained by consent from women who were undergoing premature labor and delivery. The protocol for the procurement of these cells was reviewed and approved by the IRB at Abbott Northwestern Hospital in Minneapolis, MN. The cells were maintained in Dr. Low's lab prior to use. The cells were cultured in the Dulbecco's modified Eagle medium (DMEM) and MCDB‐201(Sigma) mix with supplements including fetal bovine serum (Invitrogen), L‐ascorbic‐acid‐2‐PO4 (Sigma), dexamethasone (Sigma), insulin–transferrin–selenium media supplement (Sigma), linoleic acid/bovine serum albumin (Sigma), and epidermal growth factor (R&D Systems), and recombinant human basic fibroblast growth factor (R&D Systems).

Cell viability was accessed by cell membrane integrity. Trypan blue exclusion assay and a Countess cell counter (Thermo Fisher Scientific) was used. In addition, fluorescent Hoechst/propidium iodide (PI) staining was used.

##### Droplet Printing

CPA solution of 2 m PG and 1 m trehalose was prepared in DMEM culture medium. Sonication was used to facilitate dissolution of trehalose. A printing robot (Fisnar 5200N) and a high precision pressure dispenser (Nordson EFD) with programmable pressure pulse were used. CPA solution was loaded into a syringe connected to the pressure dispenser. Printing tip with various inner diameters was connected to the bottom of the syringe. A high‐speed camera (nac Image Technology) was employed to record the droplet printing process. To measure the volume of printed droplet, one droplet was printed onto a beaker located on a scale (Sartorius CP225D, 0.01 mg resolution) and the final weight increase was recorded. 20 replicates (*n* = 20) were performed for each printing condition. The density of the CPA solution (i.e., 1.14 g mL^−1^) was calculated using the weight of 10 mL CPA solution. The weight of printed droplet was divided by the density to calculate the droplet volume for each printing condition. A DSLR camera Nikon D750 was used to take the photos of the droplets.

##### Droplet Vitrification

Droplets in different sizes were printed directly into LN_2_. Due to Leidenfrost effect, the droplet may first float on the LN_2_ and eventually sink. The levitation time (i.e., before droplet sank into LN_2_) was recorded. In the case of printing droplet to a cryogenic copper dish, copper foil (≈200 µm thickness) was folded into a dish‐like shape so it could float on LN_2_. A wicking material (i.e., Kimwipe) was placed on the copper surface. Droplet was then printed onto the cryogenic copper dish for vitrification. CPA solutions consisting of 1 m trehalose and PG (various PG concentrations including 1, 1.5, 2, 2.5, 3, and 3.5 m) were tested to compare the above mentioned vitrification methods.

##### X‐Ray Diffraction

Rigaku MSC Micromax 007 X‐ray generators and R‐axis IV++ image plate detectors were used. One droplet was placed into the PTFE tubing with inner diameter of 1.3 mm in LN_2_. The PTFE tubing was then quickly moved to and mounted to the goniometer and stayed within a stream of cold nitrogen ≈ −170 °C. X‐ray diffractograms were collected with 30 s exposure to the X‐ray beam.

##### Raman Spectroscopy

CPA droplets (1, 2, and 4 µL) were printed onto cryogenic copper dish. In addition, 4 µL water droplet was included as a negative control (i.e., crystallization). To inhibit recrystallization during Raman measurement, droplets were placed on the cryogenic copper dish which was surrounded by LN_2_. Witec Alpha 300R confocal Raman microscope with UHTS300 spectrometer and DV401 CCD detector was used. Laser wavelength was 532 nm.

##### Cooling and Convective Warming Rates Measurement

A type T fine gage bare wire thermocouple with 50 µm wire diameter and a USB oscilloscope were used to collect the temperature profile.^[^ ^46^ ^]^ For the case of printing onto the cryogenic copper dish, the thermocouple junction was positioned at various distances from the surface of copper dish. Droplets of various sizes (1, 2, 4, 8, 16, and 32 µL) were printed onto the thermocouple such that the temperature profile at the bottom, center, or top of the droplet could be recorded. Images were taken to confirm the position of the thermocouple. Next, the vitrified droplet along with the thermocouple was dropped into unloading solution at room temperature for convective warming. Temperature profile during convective warming was recorded. Temperature zone from −20°C to −140°C was used to calculate the cooling rate and convective warming rate.

##### Cooling and Warming Rates Modeling

Comsol and Monte Carlo simulations were used. To model the cooling rate of the droplet printed onto the cryogenic copper dish, high‐speed camera recording revealed that it took ≈50 ms for the droplet to be printed onto the copper surface. Based on the measured temperature profile using a thermocouple (Figure 3), the entire cooling process took >1 s, therefore the temperature change during droplet printing was neglected. The governing equation used to solve the cooling temperature profile is

(1) $$\rho C_{p}\;\frac{\partial T}{\partial t}=\;k\nabla^{2}T$$

The initial temperature of the droplet and copper dish were set to 20 °C and −196 °C, respectively (Figure S7, Supporting Information). Convective heat flux was set as the boundary condition between the droplet and cold nitrogen vapor (−170 °C). The natural convective heat transfer coefficient was set to be 100 W m^−1^ K^−1^.^[^ ^18^ ^]^

To model the convective warming temperature profile, Equation (1) was used as the governing equation. The initial temperature of the droplet was set to −196 °C. Convective heat flux was set as the boundary condition between the droplet and rewarming medium (4 °C). Forced convective heat transfer coefficient of 500 W m K^−1^ was used.^[^ ^47^ ^]^

To model the laser warming, a customized Matlab code was used to trace the photons interaction (i.e., scattering or absorption) with the GNRs (nanoComposix Inc.) in the droplet as reported in the authors' previous publication.^[^ ^19^, ^21^ ^]^ The optical properties of GNR loaded droplets were obtained from experiments stated in previous publications.^[^ ^43^ ^]^ A distribution of specific absorption rate (SAR, W m^−3^) was generated by the Monte Carlo modeling and imported into Comsol for temperature simulation. The governing equation used to solve the laser warming temperature profile is

(2) $$\rho C_{p}\;\frac{\partial T}{\partial t}=\;k\nabla^{2}T+SAR$$

The initial temperature of the droplet was set to −196 °C. Convective heat flux was set as the boundary condition between the droplet and rewarming medium (20 °C). The natural convective heat transfer coefficient between the droplet and room temperature air was set to be 100 W m K^−1^.^[^ ^18^ ^]^

Temperature dependent density (*ρ*), heat capacity (*C*_p_) and thermal conductivity (*k*) of CPA from previous publications were used in all the simulations.^[^ ^39^, ^43^ ^]^

##### Cryoholder Fabrication and Droplet Laser Warming

The cryoholder was fabricated using a Stratasys J750 Polyjet 3D printer and the printing material called VeroClear. To laser warm the vitrified droplet, the droplet was first placed onto the cryoholder which was connected to a cryojig developed in the authors' previous publications.^[^ ^18^ ^]^ This is performed using a customized vacuum tubing device (Figure S14, Supporting Information). A valve was installed on the tubing to control the on and off of the vacuum. Briefly, a pipette tip connected to a tubing and vacuum source was used to pick up the vitrified droplets (i.e., vacuum on) and release the droplet to the cryoholder (i.e., vacuum off). The pipette tip was pre‐cooled in liquid nitrogen and the droplet transfer process was performed in liquid nitrogen. The cryojig then quickly brought the cryoholder and droplet under the laser beam for rewarming. A millisecond pulse laser with 1064 nm wavelength was used (LaserStar Technologies). The laser beam diameter was set to 4.4 mm.

##### High‐Speed Videos Recording

A high‐speed camera (MEMRECAM Q1v, nac Image Technology) was used to record the droplet printing and laser warming process. Frame rates of 4000 or 8000 frames per second were used. For laser warming, a filter screen (LaserStar Technologies) was placed in between the droplet and camera to minimize the noise from scattered laser beam.

##### Cell Cryopreservation

Cells in suspension were first centrifuged at 250 g for 5 min to form a pellet. After removing the supernatant, CPA (2m PG + 1m trehalose) with GNRs (nanoComposix, Inc) was added. The mixture was incubated on ice for 5 min and loaded into a syringe for printing onto the cryogenic copper dish. For long term storage, the vitrified droplets were collected and stored in a 50 mL conical tube in LN_2_. To calculate the yield of the printing process, cells were printed to a 1.5 mL centrifuge tube. The total numbers of cells pre‐printing and post‐printing were counted using an automatic cell counter (Thermo Fisher Scientific).

For convective warming, the cell encapsulated droplets were dropped into 4 °C 0.5 m sucrose solution directly. For laser warming, the cryojig and cryoholder brought the droplet under the laser beam. Single laser pulse was fired to rewarm the droplet. The melted droplet remained on the cryoholder due to surface tension and then transferred into 4 °C 0.5 m sucrose for CPA unloading.

To unload the CPA, cells were first exposed to 4 °C 0.5 m sucrose for 5 min. The cell suspension was centrifuged, and supernatant was removed. Ice cold fresh culture medium was added for 5 min.

For the slow freezing method, cells were loaded in a cryovial with 10% DMSO. The cryovial was transferred to the freezing contained (Mr. Frosty, ThermoFisher) which was placed in −80°C freezer overnight (i.e., cooling rate 1 °C min^−1^). The cryovial was then placed in LN_2_ for storage. For rewarming, the cryovial was agitated in a 37 °C water bath until all ice was melted. To remove the DMSO prior to membrane integrity assessment, the cell suspension was transferred to a 1.5 mL centrifuge tube, and centrifuged at 250 g for 5 min. The supernatant was removed, and fresh culture medium was added for 5 min at room temperature.

Simulation of cell volume change and intracellular CPA was performed by solving the K–K equations as follows:

(3) $$\frac{dV}{dt}=\;-L_{P}ART\left[\left(C_{s}^{e}-C_{s}^{i}\right)+\sigma \left(C_{c}^{e}-C_{c}^{i}\right)\right]$$

(4) $$\frac{dn_{c}}{dt}=\frac{\frac{1-\sigma }{2}\left(C_{c}^{e}+C_{c}^{i}\right)dV}{dt}\;+P_{s}A\left(C_{C}^{e}-C_{C}^{i}\right)$$

In the above equations, *V* is the cell volume, *A* is the surface area, *n*_C_ is the molar mass of intracellular CPA, *R* is gas constant, *T* is the absolute temperature, *σ* is the reflection coefficient, *L*_p_ is the hydraulic conductivity, *P*_s_ is the membrane permeability to CPA. *C* is the molality and superscript i denotes intracellular, e denotes extracellular, subscript C denotes permeating CPA, subscript S denoting non‐permeating solutes. For example, $C_{C}^{e}$ represents the extracellular permeating CPA molality. *L_p_* (0.16 µm per atm per min), *P_s_* (10 µm per min) and *σ* (0.81) were used for human umbilicao cord blood cells; and *L_p_* (0.11 µm per atm per min), *P_s_* (2.7 µm per min) and *σ* (0.58) for human dermal fibroblast based on the literature.^[^ ^48^, ^49^ ^]^

##### Statistical Analysis

Data were tested for normality using the Shapiro–Wilk normality test. Viability of the recovered cells post treatment was normalized to the viability of untreated cells. All experimental data were presented as mean ± SD. Sample size (*n*) for experimental data was included in the figure captions. A paired one‐way ANOVA and Tukey's post hoc were used for the statistical analysis of different treatment groups shown in Figure 5; and Figure S17, Supporting Information. The *p* values >0.05 were considered statistically non‐significant (*ns*). The *p* values <0.05 were considered statistically significant, with different levels representing: * *p* < 0.05, ** *p* < 0.01, *** *p* < 0.001. All analyses were performed in GraphPad Prism software.

## Conflict of Interest

The authors declare no conflict of interest.

## Supporting information

Supporting Information

Supplemental Movie 1

Supplemental Movie 2

Supplemental Movie 3

## Data Availability

Research data are not shared.
