# Supplementary material for: Conduction Cooling and Plasmonic Heating Dramatically Increase Droplet Vitrification Volumes for Cell Cryopreservation
Source: Adv Sci (Weinh). 2021 Apr 10;8(11):2004605. doi: 10.1002/advs.202004605 (PMC8188207; doi:10.1002/advs.202004605)
Supplement: Supplementary file 1 — Supporting Information [file ADVS-8-2004605-s002.pdf]

© 2021 Wiley-VCH GmbH

Supporting Information

for *Adv. Sci.,* DOI: 10.1002/advs.202004605

Conduction Cooling and Plasmonic Heating Dramatically Increase Droplet Vitrification Volumes for Cell Cryopreservation

*Li Zhan, Shuang-Zhuang Guo, Joseph Kangas, Qi Shao, Maple Shiao, Kanav Khosla, Walter C. Low, Michael McAlpine, John Bischof**

Supporting Information

Conduction cooling and plasmonic heating dramatically increase droplet vitrification volumes for cell cryopreservation

*Li Zhan, Shuang-Zhuang Guo, Joseph Kangas, Qi Shao, Maple Shiao, Kanav Khosla, Walter C. Low, Michael McAlpine, John Bischof**

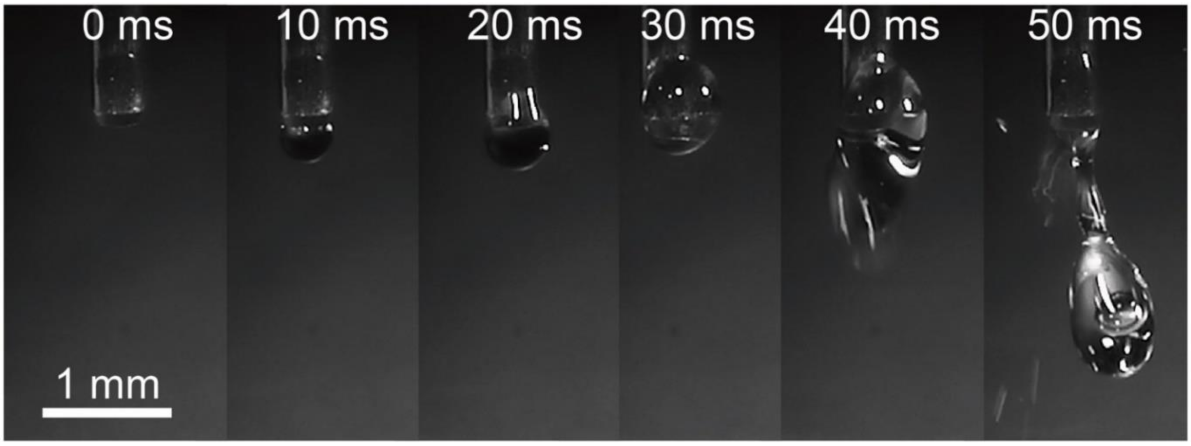

Figure S1. Images of the droplet printing process at different time. Printing tip inner diameter is 0.41 mm, printing pressure is 8 kPa, printing time is 50 ms.

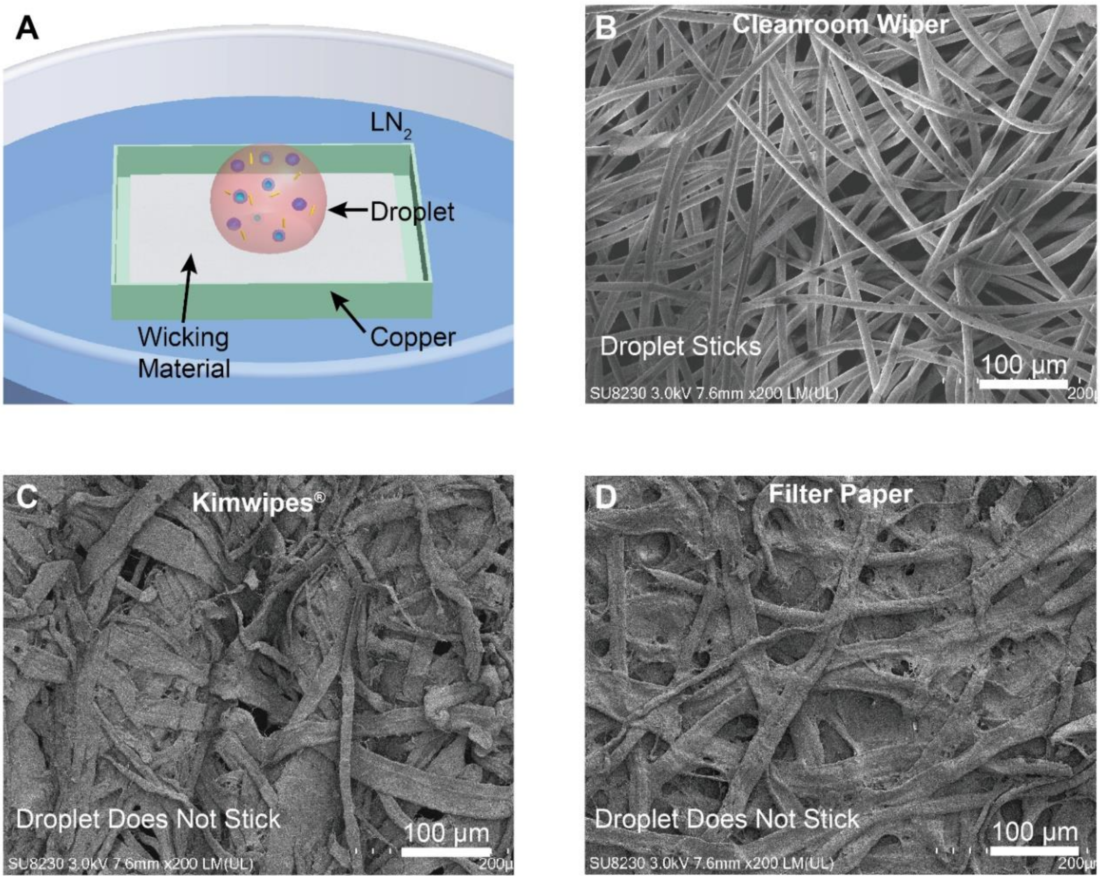

Figure S2. Various materials were used to wick the condensed liquid away from the surface of the copper dish. (A) Without the wicking material on the copper dish, nitrogen and oxygen (boiling point -183 °C) will condense and eventually lead to the "Leidenfrost" boiling. (B) When cleanroom wipe was used, the vitrified droplet stuck to the wipe and cannot be removed. (C-D) When Kimwipes® and filter paper were used, the vitrified droplet did not stick and can be easily removed.

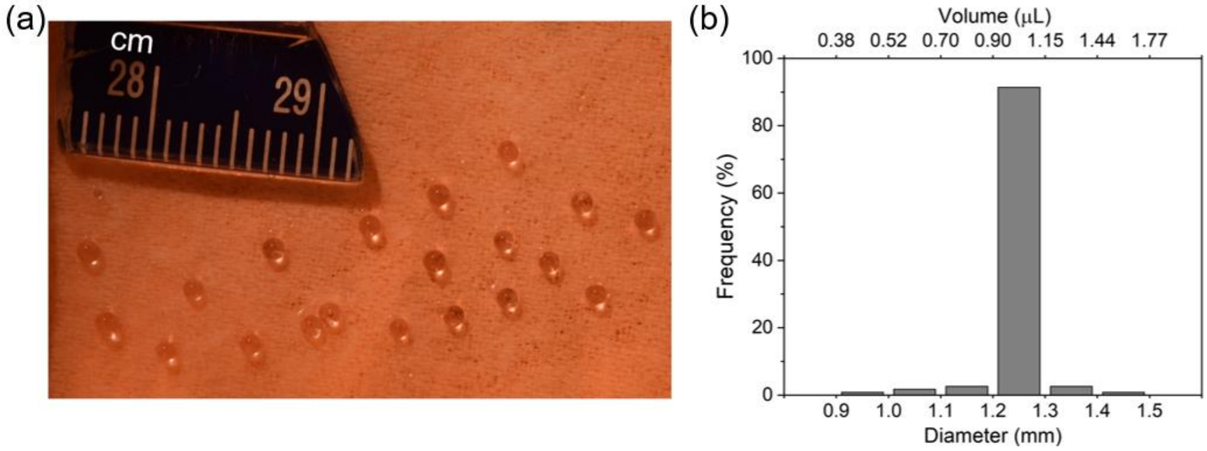

Figure S3. Droplet size distribution. (a) Representative picture of printed and vitrified droplets on a copper dish with Kimwipes® as the wicking material. The major unit for the ruler is cm and the minor unit is mm. (b) The diameters of the vitrified droplets were measured to obtain the size distribution. One microliter droplets were printed, and 116 droplets were counted.

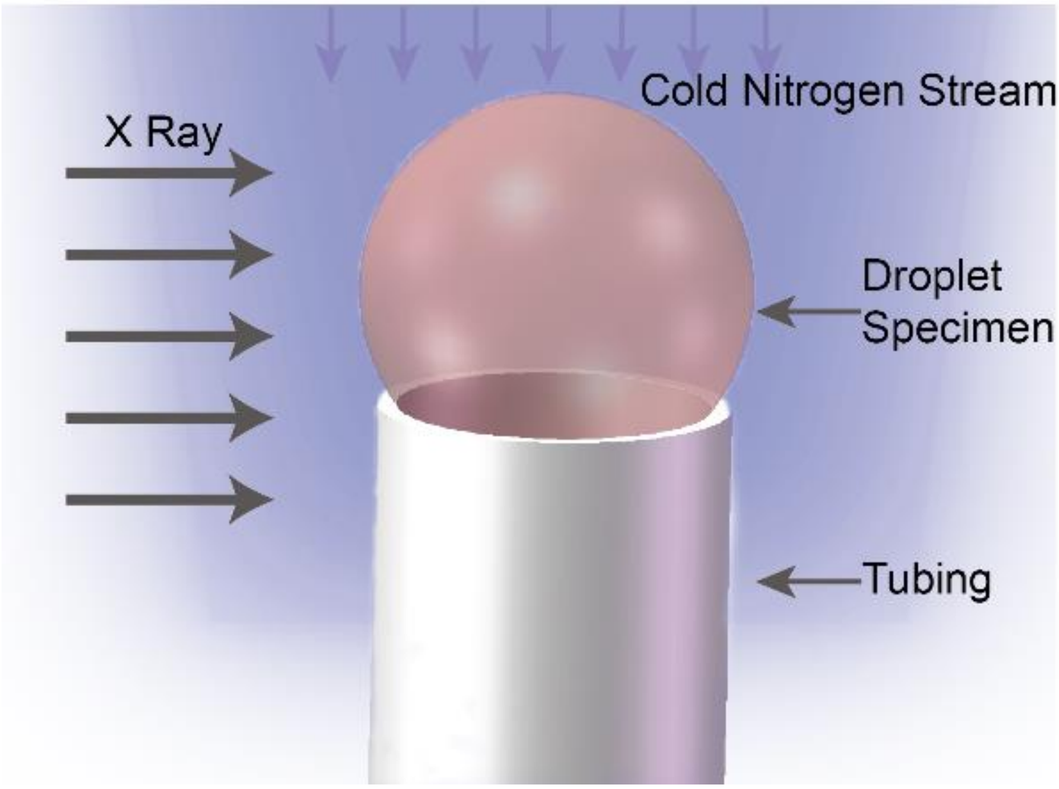

Figure S4. Schematic of X-ray diffraction measurement of droplets around -170 °C. A tubing was used to hold the droplet under cold nitrogen stream.

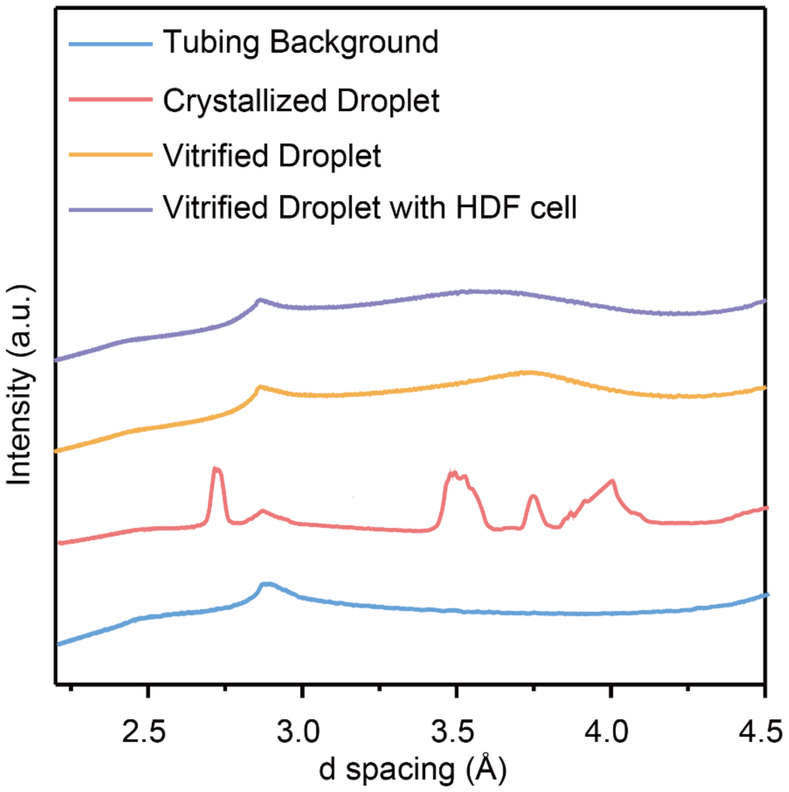

Figure S5. Representative d spacing of the X-ray diffraction measurement. The tubing material has a peak at 2.8 Å as the background. Crystallized droplet showed peaks at 2.6 Å, 3.4 Å,3.6 Å,3.9 Å which are associated with hexagonal ice crystals. Vitrified CPA droplets and vitrified droplets with HDF cells showed a broad peak around 3.6 Å.

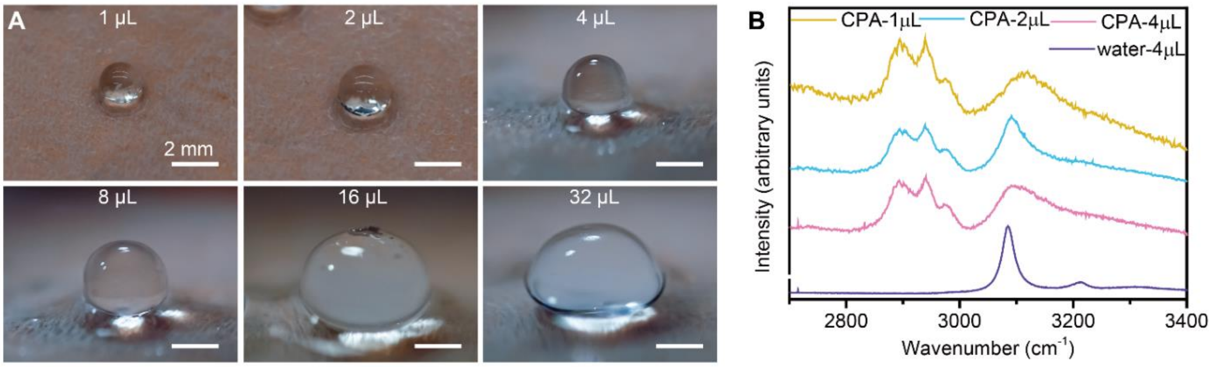

Figure S6. (A) Representative images of printed and vitrified droplets in different sizes. Scale bars are 2 mm. (B) Representative Raman spectra of CPA droplets (i.e., vitrified) and water droplet (i.e., crystallized). The peak of O-H stretching band (~ 3100 cm^{-1}) shifts toward lower wavenumber in crystallized samples.

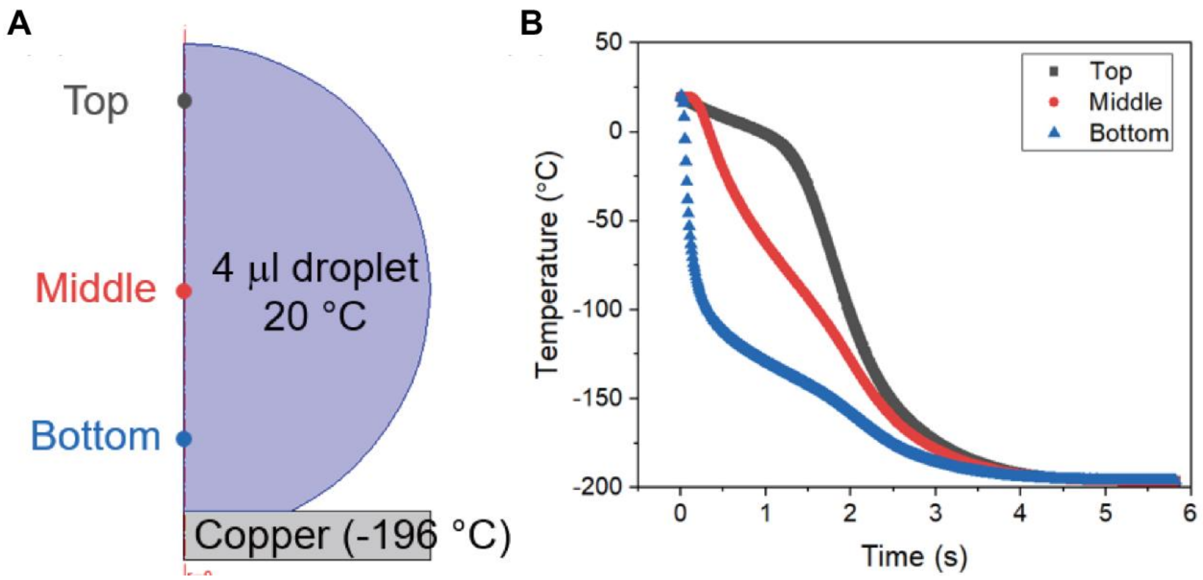

Figure S7. Simulated temperature profile of a 4 mL droplet during cooling on the copper dish. (A) Three points including "top", "middle" and "bottom" were selected to mimic the thermocouple locations in Figure 3D. The initial temperature of the droplet and copper is 20 °C and -196 °C respectively. (B) Simulated temperature of "top", "middle" and "bottom" points within the droplet.

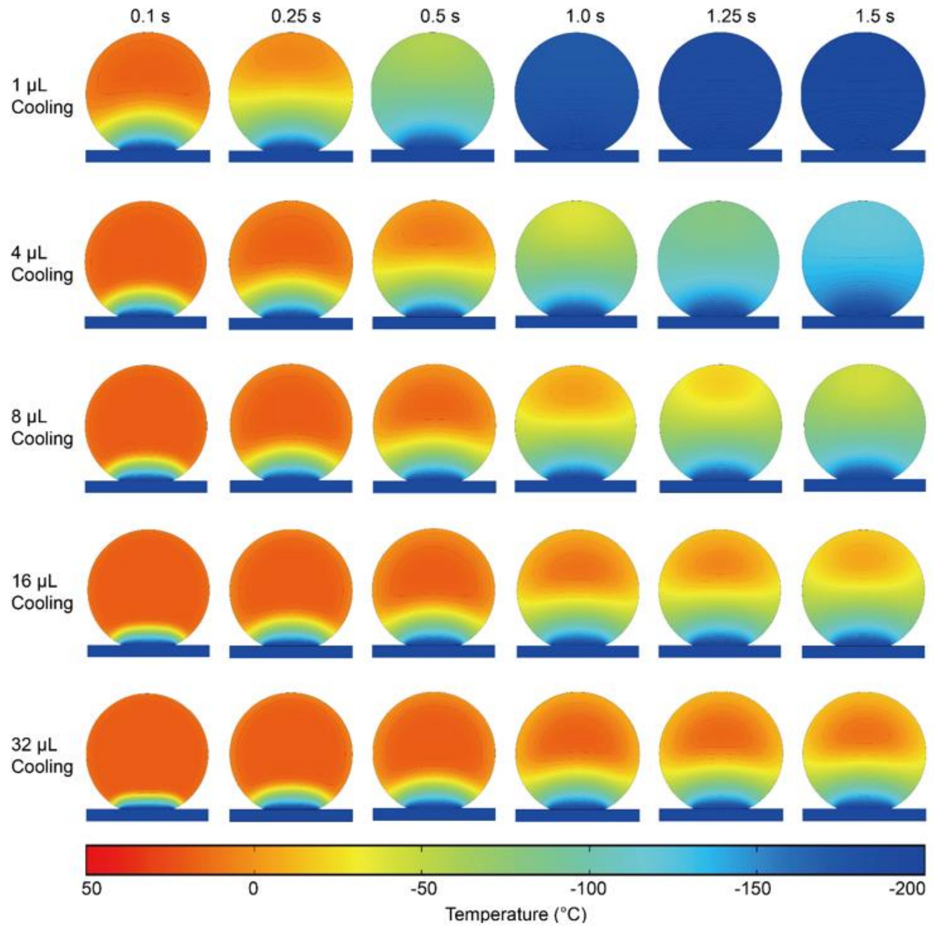

Figure S8. Simulated temperature distribution of droplets of different volumes during cooling on the copper dish. Droplets were normalized to the same visual size in the image for convenient comparison.

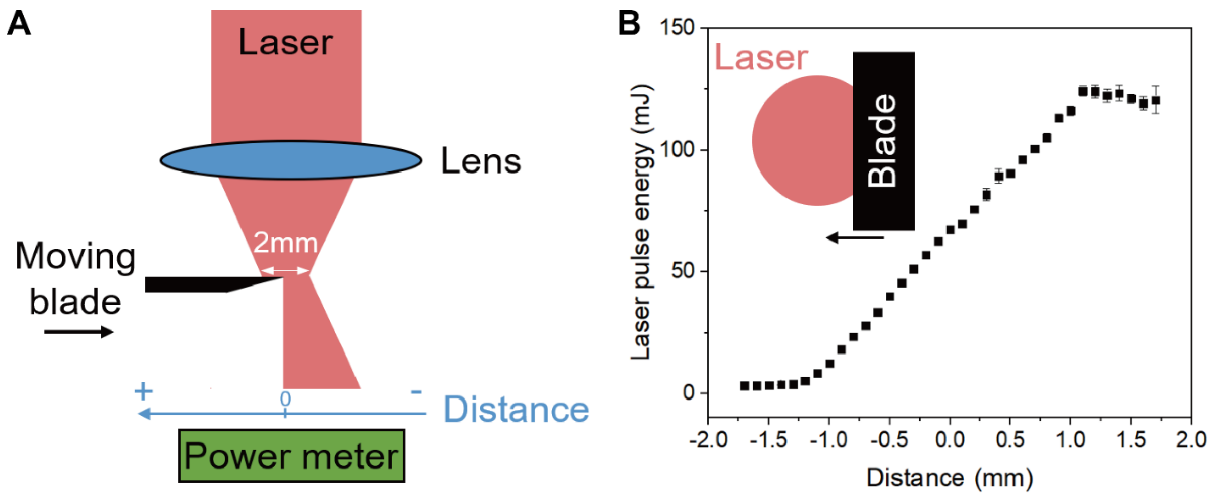

Figure S9. "Knife edge" measurement of laser beam energy distribution. (A) Schematics of the measurement setup, a blade mounted to a translational stage was used to block part of the laser beam and the power of the remaining beam was recorded. The blade distance moved was recorded along with the power transmitted. (B) The measured laser pulse energy as a function of blade distance moved (n=5). The laser beam was set to 2 mm from the pulse laser. This linear energ.y measured by the power meter indicates a top-hat laser beam (i.e., energy is uniformly distributed within the beam), instead of a Gaussian beam. Data points represent mean ± s.d.

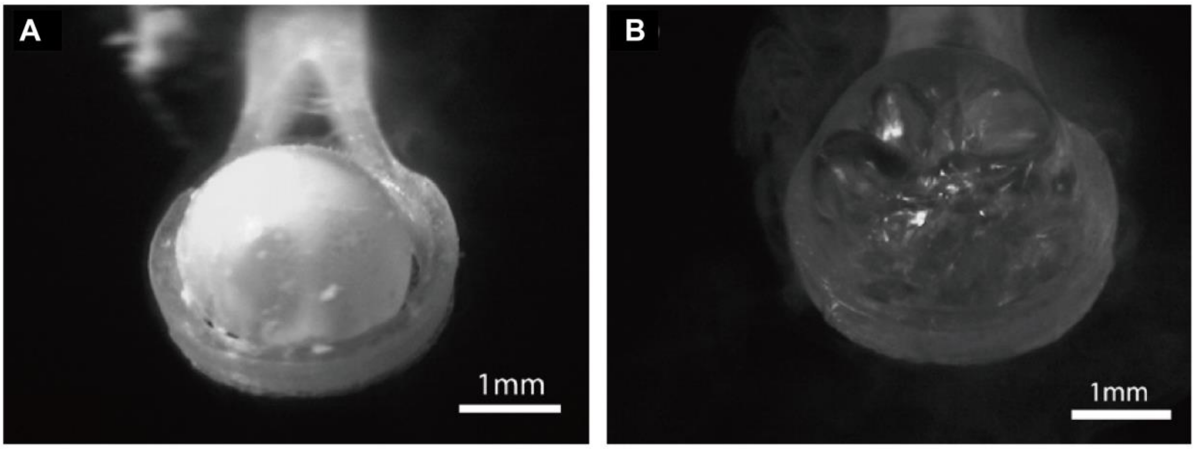

Figure S10. Example of (A) underheating (i.e., devitrification) and (B) overheating (i.e., boiling) of a 4 μL droplet during laser warming.

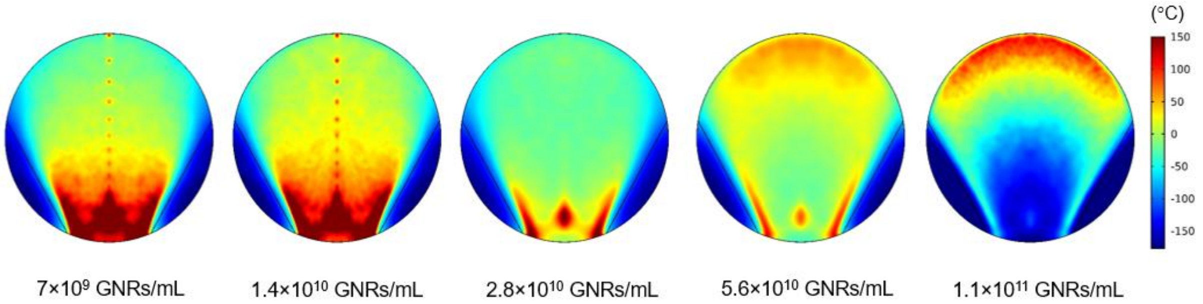

Figure S11. Simulated temperature distribution within the droplets using different GNR concentrations. As GNR concentration increases, the hot zone (i.e., localized high temperature area) moves from bottom to top within the droplet. With low GNR concentration, laser can penetrate deep into the droplet and will eventually get focused at the bottom. With high GNR concentration, most laser energy is absorbed by the GNR on the top. Ideal GNR concentration provides a relatively uniform temperature distribution.

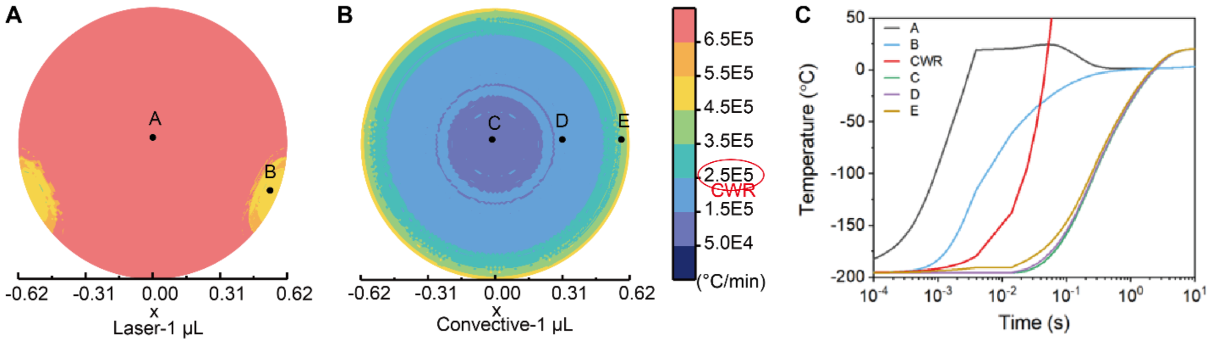

Figure S12. Droplet rewarming simulation. (A) Distribution of warming rate inside the laser warmed 1 μL droplet. (B) Distribution of warming rate inside the convectively warmed 1 μL droplet. (C) Temperature profile at representative locations inside the droplets. Critical warming rate (CWR) was set to (2.5E5) 2.5×10^5 °C/min.

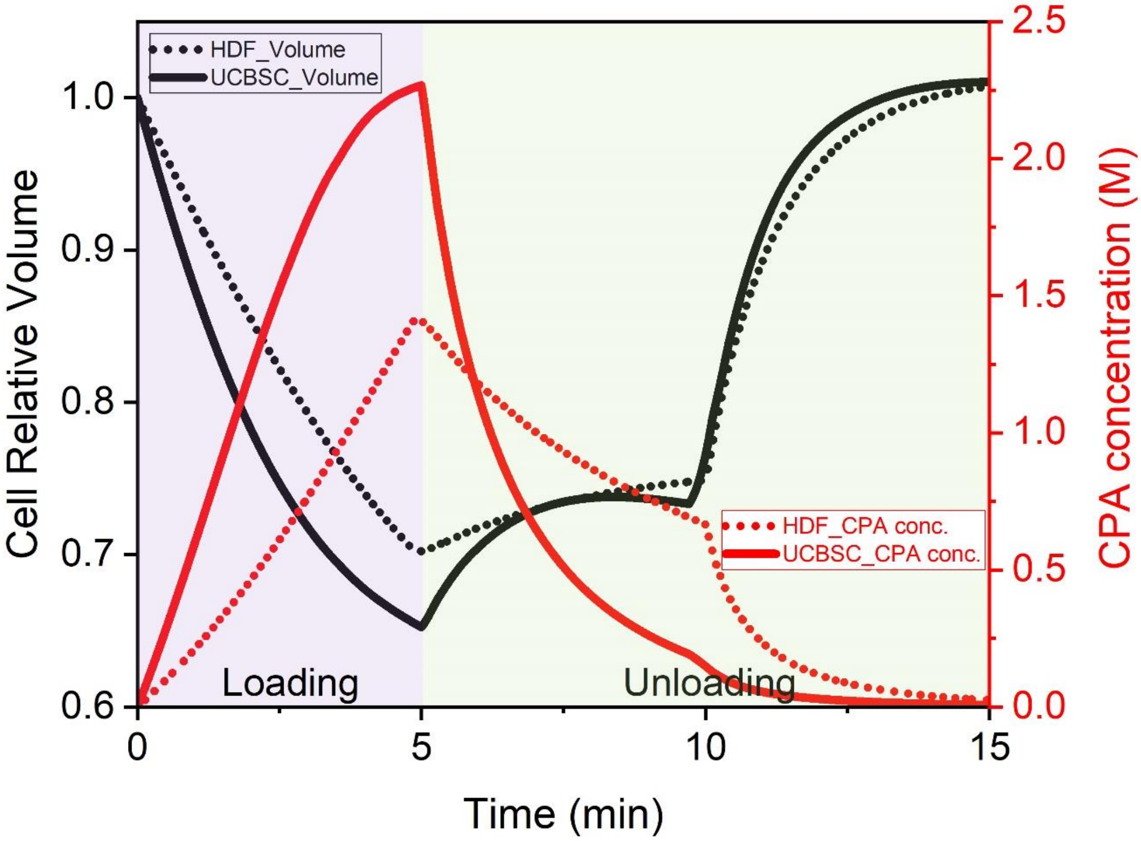

Figure S13. Simulated cell relative volume (black) and intracellular CPA concentration (red) of HDF cells (dotted lines) and UCBSCs (solid lines) during CPA loading and unloading process.

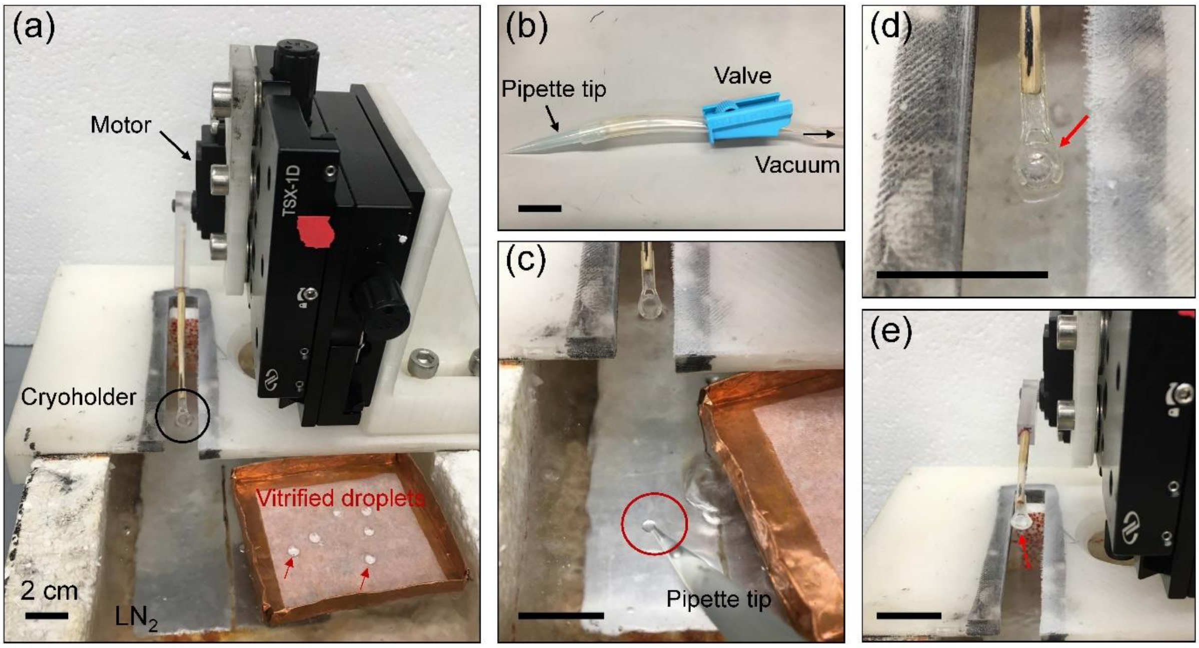

Figure S14. The process of transferring vitrified droplet from the copper dish to the cryoholder for laser warming. (a) Copper dish with vitrified droplets is first placed in a styrofoam box filled with liquid nitrogen. The cryoholder is placed in liquid nitrogen (or close to the liquid nitrogen surface) and connected to a customized cryojig which includes a motor to rapidly move the cryoholder. (b) A customized vacuum tubing device consists of a pipette tip (1 mL) on one end and a valve to control the vacuum. The other end of the tubing is connected to the vacuum source. (c) The vacuum tubing device picks up a vitrified droplet. First, the pipette tip is cooled in liquid nitrogen, then the valve is opened (i.e., vacuum on), a vitrified droplet from the copper dish is quickly picked up by the pipette tip and placed in liquid nitrogen. (d) The vitrified droplet is placed on the cryoholder by closing the valve (i.e., vacuum off). (e) The cryojig rapidly brings up the cryoholder and vitrified droplet for laser warming.

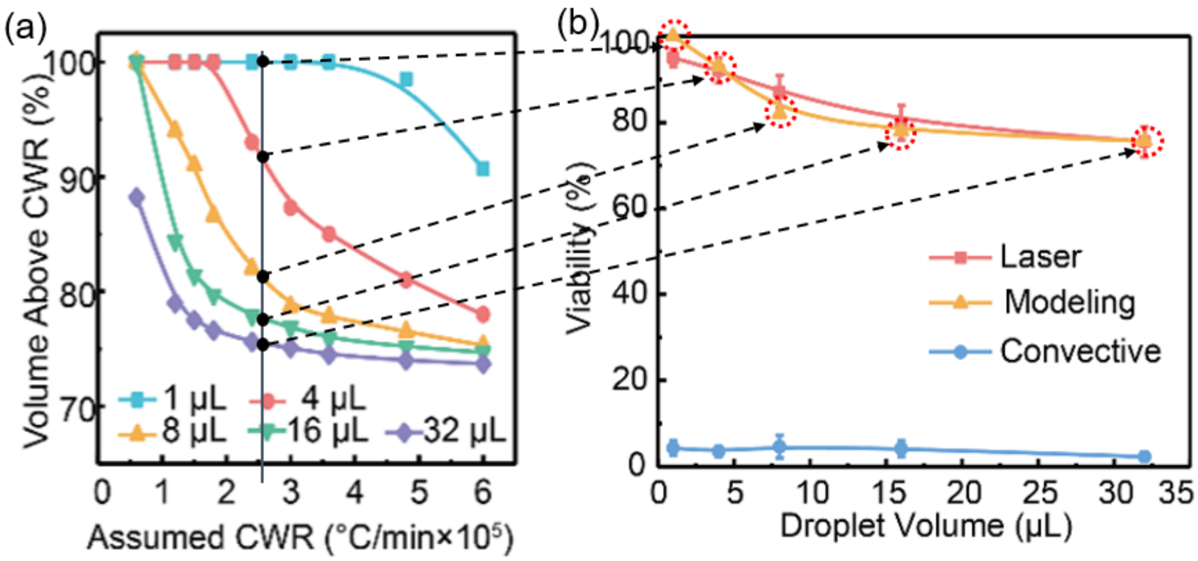

Figure S15. Estimation of CWR for 2 M PG + 1 M trehalose. (a) Simulated volume percentage of the droplet with warming rate above a certain assumed critical warming rate (CWR). Multiple droplet sizes were plotted. For a certain assumed CWR (i.e., the black verticle line), the volume percentrage above CWR are collected (i.e., the black dots). (b) The simulated volume percentage above CWR was used to predict the cell viability post laser warming. Experimental cell viability was also plotted.

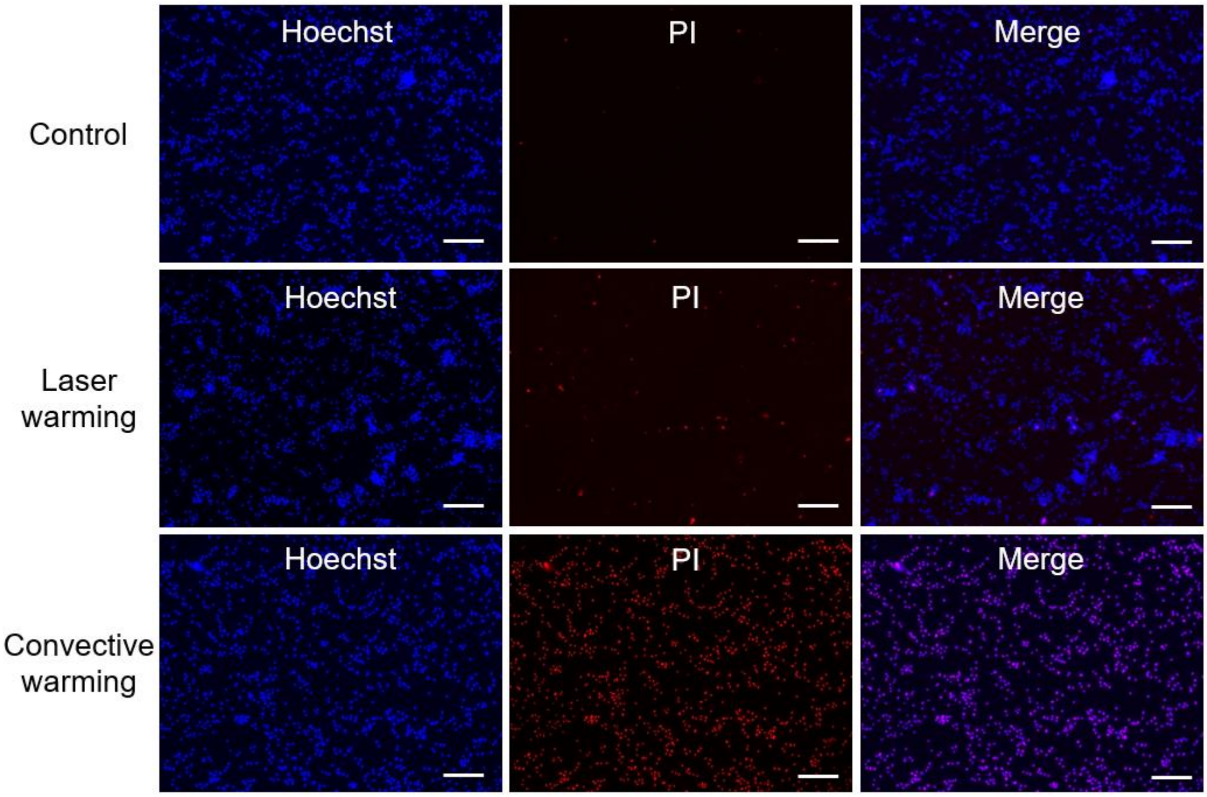

Figure S16. Hoechst/PI staining of HDF cells. Top row is control group without any treatment. Middle row is laser warming group and bottom row is convective warming group.

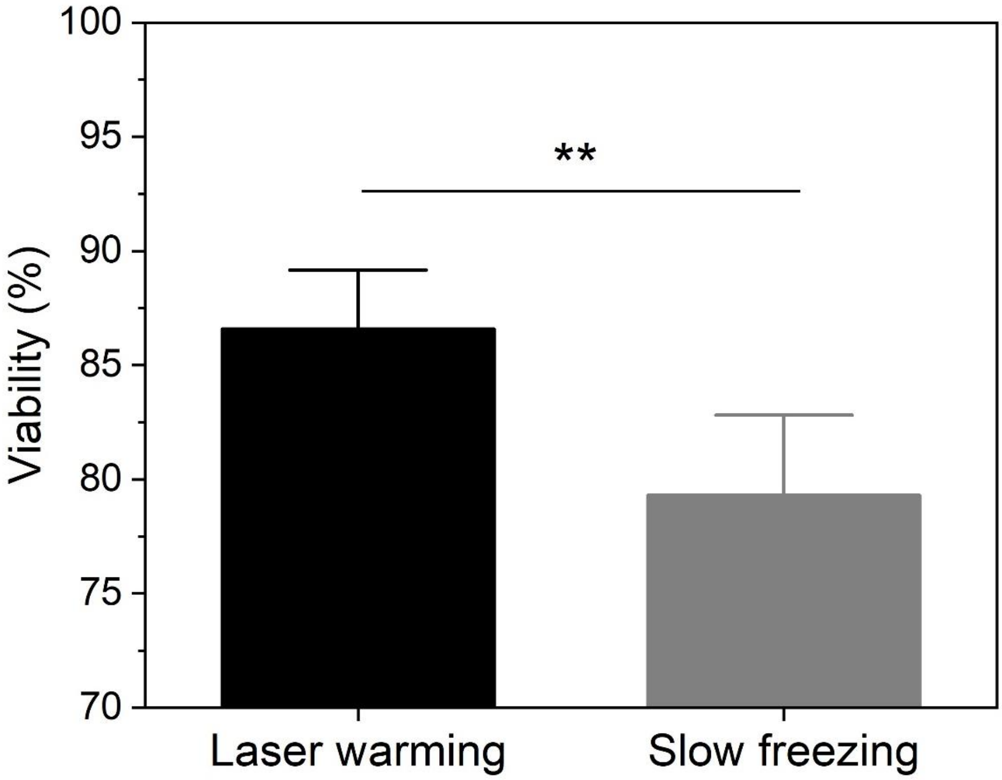

Figure S17. Viability of human umbilical cord blood stem cells using traditional slow freezing method vs droplet laser warming method developed in this work. Post cryopreservation viability is calculated as the ratio of total intact cells post thaw and total intact cells before cryopreservation. The yield of the cryopreservation approach was accounted for in the comparison. Data points represent mean ± s.d. n = 5, ** $p<0.01$. One-way ANOVA and Tukey post hoc was used.

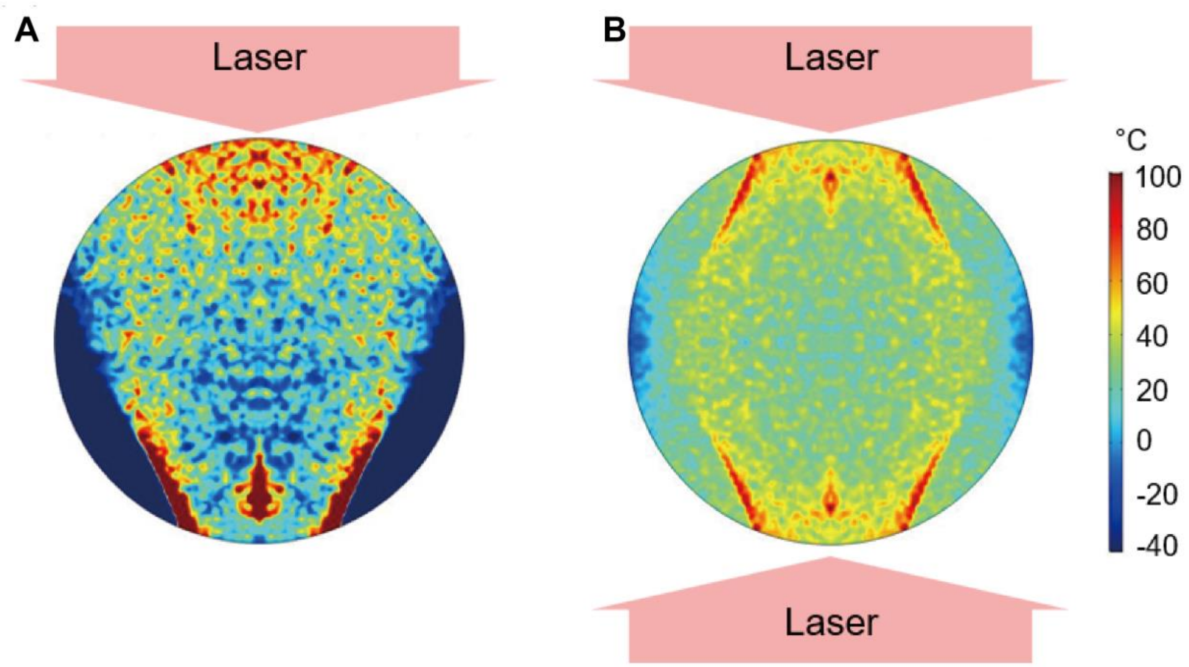

Figure S18. Simulated temperature distribution within 32 μL droplets using one laser vs two lasers. (A) Temperature distribution of the droplet when only one laser was used to rewarm the droplet. Lensing effect leads to non-uniform temperature distribution. (B) Two lasers were used to rewarm the droplet from two directions, leading to a more uniform temperature distribution.

Movie S1. Droplet printed onto a cryogenic copper dish.

Movie S2. Laser warming of a 4-microliter droplet.

Movie S3. Laser warming of a 32-microliter droplet.
